# Polyphyly of nuclear lamin genes indicates an early eukaryotic origin of the metazoan-type intermediate filament proteins

**DOI:** 10.1038/srep10652

**Published:** 2015-05-29

**Authors:** Martin Kollmar

**Affiliations:** 1 Group Systems Biology of Motor Proteins, Department of NMR-based Structural Biology, Max-Planck-Institute for Biophysical Chemistry, Göttingen, Germany

## Abstract

The nuclear lamina is a protein meshwork associated with the inner side of the nuclear envelope contributing structural, signalling and regulatory functions. Here, I report on the evolution of an important component of the lamina, the lamin intermediate filament proteins, across the eukaryotic tree of life. The lamins show a variety of protein domain and sequence motif architectures beyond the classical α-helical rod, nuclear localisation signal, immunoglobulin domain and CaaX motif organisation, suggesting extension and adaptation of functions in many species. I identified lamin genes not only in metazoa and Amoebozoa as previously described, but also in other opisthokonts including Ichthyosporea and choanoflagellates, in oomycetes, a sub-family of Stramenopiles, and in Rhizaria, implying that they must have been present very early in eukaryotic evolution if not even the last common ancestor of all extant eukaryotes. These data considerably extend the current perception of lamin evolution and have important implications with regard to the evolution of the nuclear envelope.

Eukaryotes are distinguished from prokaryotes by having a nuclear envelope (NE) separating the genetic material from the cytoplasm. The NE is composed of an inner and outer nuclear membrane that are connected at nuclear pore sites and separated by perinuclear space. Many types of channels and pores allow the transfer of proteins and molecules between nucleus and cytoplasm^1^,^2^. Defects in proteins associated with the NE are linked to many human diseases^3^,^4^,^5^,^6^. A dense protein meshwork, primarily composed of A- and B-type lamins in vertebrates, is associated with the nuclear face of the inner nuclear membrane^7^,^8^,^9^. Although lamin genes are thought to be restricted to metazoans^9^,^10^,^11^, fibrous protein networks associated with the NE have also been reported for protozoans like the amoebozoans *Amoeba proteus*^12^ and *Entamoeba invadens*^13^, the alveolate *Gregarina melanopli*^14^, the euglenophyte *Euglena gracilis*^15^, the diplomonad *Giardia lamblia*^13^ and the parabasalid *Trichomonas vaginalis*^13^. While the evidence for lamin-like structures in these species are electron micrographs and Western blot analyses, a nucleoskeleton protein sequence similar to lamins has recently been identified in the amoebozoan *Dictyostelium discoideum*^16^.

Lamins belong to the multigene family of metazoan intermediate filament (mIF) proteins comprising the nuclear lamins (Type V mIF proteins) and several types of cytoplasmic mIFs such as the vertebrate trichocyte and epidermal keratins (Type I and Type II), desmin, vimentin, peripherin, and glial fibrillary acidic proteins (Type III), and neurofilaments and α-internexin (Type IV). This set of mIF proteins has been established already 30 years ago^17^ but is continuously expanding mainly due to the possibilities of genome sequencing. Phakinin, filamin, tanabin, nestin and synemin are restricted to vertebrates and usually referred to as Type VI mIF proteins^18^. Invertebrate mIF proteins are commonly not classified into specific subtypes although they show a similar extend of unique additions to the α-helical core domain^19^. In some basal animal species, unique mIF proteins have been identified localizing to specific cellular structures such as nematocilin^20^. In addition to these cannonical intermediate filament proteins, coiled-coil and other repeat containing proteins have been reported to be part of networks of 5–10 nm filaments in protists: the euglenozoan-specific articulins^21^, the tetrins^22^,^23^ and epiplasmins^24^ currently restricted to Ciliophora, and the alveolins (also called IMC proteins in apicomplexans), which are restricted to alveolates^25^,^26^. The experimental observation of nuclear lamina in protozoa and the wealth of mIF diversity provoked me to search the available genome data to redraw the history of the lamins.

## Results

### Identification of metazoan intermediate filament proteins

**Figure 1:**
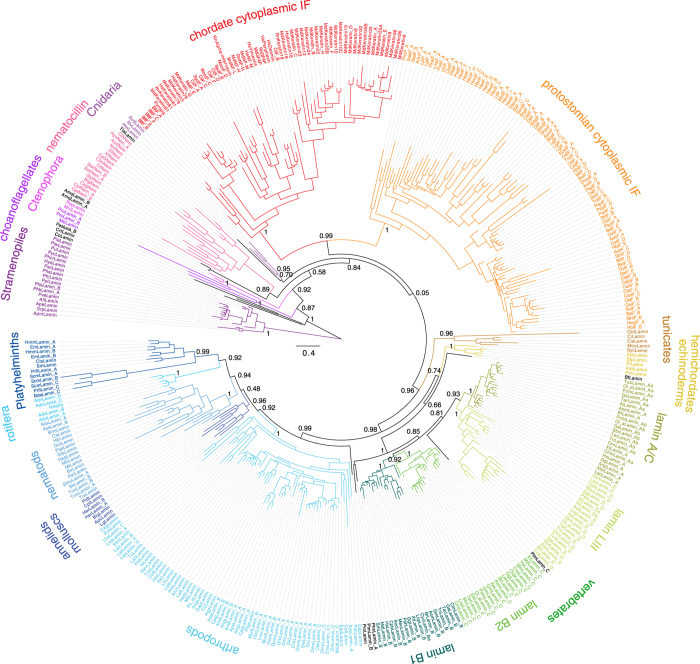
Phylogeny of the intermediate filament proteins. Maximum-likelihood topology generated under the JTT + Γ model as implemented in FastTree showing branch lengths for 232 lamins, 16 nematocilins, and 145 cytoplasmic mIF proteins. To obtain a representative dataset for subtype classification and visualization, CD-Hit (90% idenity) was used, and the amoebozoan lamins as well as highly variable regions were removed manually. Support for major branchings indicating the grouping of the different types of IF proteins is given as likelihood bootstrap. In general, the major branches such as the groupings of the protostomian and chordate cytoplasmic IFs are strongly supported. However, groups with low taxonomic sampling are sometimes less supported and branch differently in trees generated from different datasets (Supplementary Fig. S2). For instance, the grouping of the two poriferan lamins from *Amphimedon queenslandica* and the single placozoan lamin from *Trichoplax adhaerens* varies between trees. The scale bar represents the estimated number of amino acid substitutions per site.

In order to identify lamin proteins I examined most of the publically available genome assemblies of eukaryotes^27^ using sequence similarity searches followed by manual inspection of the genomic DNA sequences^28^,^29^. In addition, EST/cDNA databases and transcriptome shotgun assembly data were analysed for species for which genome data are not available. While lamin genes are conserved within closely related taxa (e.g. within vertebrates, within Diptera, or within Amoebozoa), TBLASTN hits in other species cannot always be distinguished for lamins and mIF proteins. Therefore, for most genomes I assembled all search hits and annotated the resulting genes while correcting the predicted sequences based on the multiple sequence alignment (Supplementary Data S1, Supplementary Fig. S1). Protein sub-family relationships have finally been resolved by reconstructing phylogenetic trees using Neighbour Joining and the Maximum likelihood approaches (Fig. 1, Supplementary Fig. S2). The tree topologies of the major branches were almost identical, independently of the tree reconstruction method and the removal of redundant sequences and divergent regions. The gene trees are consistent with the latest species phylogenies including all known whole-genome duplication events, except for the chordate *Oikopleura dioica* lamin, the amoebozoan lamins, and a group of divergent flatworm (Platyhelminthes) lamins that always group together with the nematocilins. However, if either the amoebozoan or both the *O.dioica* and flatworm lamins were excluded from the tree reconstruction, the respective remaining lamins grouped in the order that would have been expected from the species phylogeny (the amoebozoan lamins outside the metazoans, and the *O.dioica* and flatworm lamins to the other chordate and flatworm lamins, respectively; Supplementary Figs. S3, S4 and S5). These findings strongly indicate branch-attraction effects between these groups of lamins. Lamin and mIF family proteins were identified in many major branches of the eukaryotes, Stramenopiles, Rhizaria, Amoebozoa and Opisthokonts, but only metazoan species contained several distinct subtypes. Gene duplicates have not only been found in *Drosophila* and vertebrates but also outside metazoans. Fragments of lamin and IF proteins in plant and rhodophyte EST/cDNA libraries could not be confirmed in any of the respective genome assemblies but closely grouped to oomycete, nematode and arthropod homologs, indicating considerable contamination of the cDNA data (Supplementary Table S1).

### Polyphyly of the lamins

The phylogenetic trees showed the metazoan cytosplamic intermediate filament protein families to form three well-supported distinct groups. The vertebrate Type I to Type IV chains and mIF proteins from cephalochordates and tunicates form a chordate-specific group. The lophotrochozoan (Platyhelminthes, molluscs, annelids, arthropods and nematods) mIF proteins form a sister clade to the chordate mIF group, and the nematocilins (including the *Nematostella* nematocilin^20^, which is also known as cytovec^30^) group together outside the bilaterian proteins. Surprisingly, in all reconstructed trees the lamins were polyphyletic independent of the branch that was used to root the tree. The polyphyly might be caused by several reasons: i) The tree reconstructions are not reliable, ii) the definition of lamin needs to be adjusted, or iii) the polyphyly is part of the evolution of the lamin proteins. There are several indications that the tree reconstructions are indeed very reliable. If all intermediate filament proteins analysed were regarded as lamins, the phylogenetic trees would exactly represent the species’ tree with lamin duplications early and later in metazoan evolution. Any other possible, but not observed, topology such as a different branch point for the nematocilins or the cytoplasmic mIF proteins would either not be consistent with the species phylogeny, imply an ancient duplication with subsequent massive loss of nematocilin and/or cytoplasmic mIF proteins in many branches, or suggest the independent invention of nematocilin and/or cytoplasmic mIF proteins by many species. All these latter possibilities seem extremely non-parsimonious.

### Lamin domain architecture

**Figure 2:**
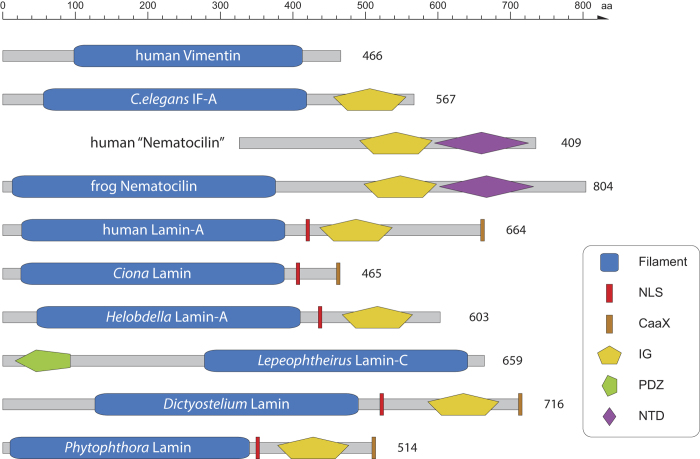
IF protein domain architecture. The schemes show the spectrum of observed domain organisations in lamins and selected mIF proteins.

Lamins are characterised by an N-terminal α-helical rod domain followed by a nuclear localisation signal (NLS), an immunoglobulin (IG) domain and a C-terminal CaaX motif (Fig. 2). Known exceptions were the ascidian lamins^31^ and one of the *Drosophila* gene duplicates (*Dm*Lamin-C)^32^ that miss the IG domain and the CaaX motif, respectively. Although the extended domain structure is present in most lamins, the data show that the only domain present in all lamins is the α-helical rod domain (Fig. 2). Like in other eukaryotic multi-domain protein families (e.g. coronins^33^, myosins^34^, kinesins^35^, WASP family proteins^36^), loss and gain events of domains and motifs led to diverse architectures. For example, in addition to the ascidian lamins, one of the three *Glossina morsitans* (Savanna tsetse fly) lamins and one of the three *Acyrthosiphon pisum* (pea aphid) lamins miss the IG domain implying that the IG domain loss happened independently in at least two major branches, and also independently in two species of the same branch. Homologs to the *Drosophila* lamin-C missing the CaaX motif are present in all Brachycera but no other Diptera, indicating the brachyceran origin of this lamin subtype. In addition, the CaaX motif has independently been lost in the two lamins of the annelid *Helobdella robusta*, in the *Capitella teleta* lamin, in one lamin of *Adineta vaga*, and in the *Salpingoeca* (a choanoflagellate) lamins. The lamins of the sponge *Amphimedon queenslandica* and the other *Adineta vaga* lamin miss both the IG domain and the CaaX motif. Most surprisingly, I identified lamins in the salmon louse *Lepeophtheirus salmonis* (*Lhs*) and in *Eurytemora affinis* that miss the entire C-terminal tail (NLS, IG domain, CaaX motif), and instead have long N-terminal extensions with a PDZ signalling domain in the *Lhs*Lamin-C. In total, however, these lamin domain architecture variations are rather the exception than the rule. Interestingly, the lamins of the most divergent species, the choanoflagellate *Monosiga brevicollis*, the Ichthyosporea *Capsaspora owczarzaki*, the amoebae, and the oomycetes (stramenopiles), have the classical domain architecture (Fig. 2). In contrast to the lamins, the domain architectures of the cytoplasmic mIF proteins are conserved within subtypes. The nematocilins have the α-helical rod domain followed by the IG domain and a conserved C-terminal region, which I suggest to name *nematocilin-tail-domain* (NTD, Supplementary Fig. S6). The protostomian cytoplasmic mIF proteins consist of α-helical rod and IG domains with long N-terminal extensions in some nematode mIFs. The chordate cytoplasmic mIF proteins share the α-helical rod domain with mIF type-specific N- and C-terminal additions. Thus, what makes a lamin a lamin? As long as experimental data is missing for most of the predicted sequences, the strongest criterions are sequence similarity of the α-helical rod and IG domains, and conservation of the classical domain architecture. The lamins mentioned above, that miss parts of the classical domain organisation, show higher sequence similarity to the other lamins than to the cytoplasmic mIFs. Accordingly, they group closest to other lamins of the same species or to lamin homologs of respective closely related species in the phylogenetic trees. For example, the unusual salmon louse lamin *Lhs*Lamin-C has most probably been derived by gene duplication of either *Lhs*Lamin-A or *Lhs*Lamin-B, followed by loss of the NLS, IG domain and CaaX motif, and gain of the N-termial PDZ domain (Fig. 1). Gene structure (intron position) conservation has also been proposed and used for intermediate filament protein classification^37^,^38^,^39^,^40^. It has been found that all nine intron positions of the *Nematostella* lamin gene are conserved in human lamin genes^41^. In contrast, the five *C.elegans* lamin intron positions and the two *Drosophila* lamin-A (Dmo) introns are not shared with chordates^40^,^41^. However, these findings are due to the low taxonomic and sequence sampling. For example, the waterflee *Daphnia* and the nematode *Pristionchus* lamin genes have up to 18 introns, including the *C.elegans* lamin positions, supporting earlier findings of extensive intron loss compared to intron gain in eukaryotes^42^,^43^. Thus, models for lamin and mIF gene evolution based on nine introns^40^,^44^ might be valid for vertebrate genes but cannot be further generalized.

In addition to missing domains and sequence motifs as described, many lamins contain gaps and/or insertions in the α-helical rod domain (Supplementary Fig. S1). These gaps/insertions might either be species-specific (e.g. a *Petromyzon* lamin-C specific insertion in the L1 region) or conserved throughout a taxonomic branch (e.g. a Platyhelminth-specific deletion and a Ctenophora-specific insertion in the 1b region, Supplementary Fig S1). In all cases, insertions and deletions in coiled-coil regions are either 7 or 11 residues long or multiples thereof, indicating the overall preservation of the coiled-coil structure.

### Eukaryotic intermediate filament evolution

**Figure 3:**
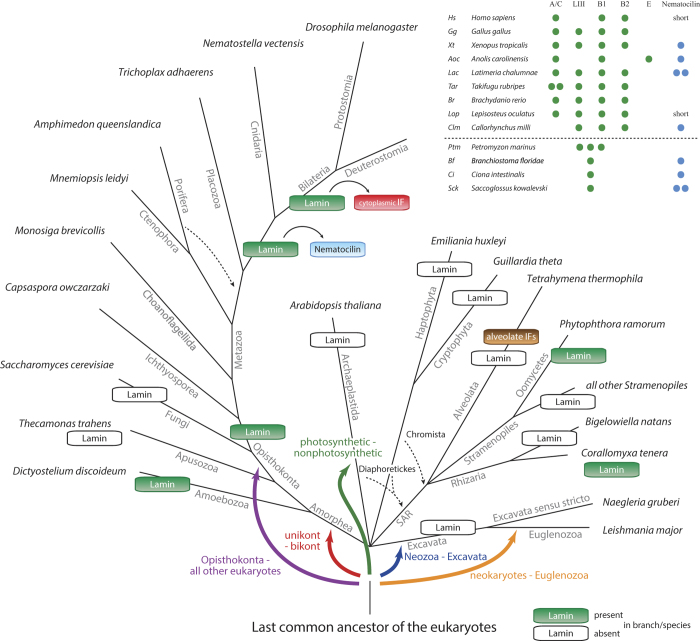
Lamin and canonical metazoan-type IF protein evolution across eukaryotes. The schematic tree represents the most commonly agreed phylogeny of the eukaryotes. Branches, in which lamin/mIF genes were not identified, were added based on the consensus of recent literature. Dashed lines indicate alternative placing of branches and coloured arrows illustrate alternatives for placing the root of the eukaryotes. The alveolate IFs include tetrins, alveolins, articulins, and epiplasmins. The inlet table lists the lamin and nematocilin inventories of representative deuterostomian species.

With these results at hand the most likely way how to explain the radiation of the lamin family is to place the root of the tree at the taxonomically earliest diverging group, the oomycete lamins (Fig. 1 and Fig. 3). Next to the ooymcetes, the Rhizaria *Corallomyxa* lamin, the *Capsaspora* lamin and a lamin of unknown origin, which was found as contamination in a *Festuca* EST dataset, group together followed by the amoebozoan and choanoflagellate lamins. While the *Capsaspora* lamin always groups between the oomycete and choanoflagellate lamins, the taxonomic sampling of this group of species is not high enough to exactly resemble the species phylogeny. However, all non-metazoan lamins invariably group outside the metazoan lamins and cytoplasmic mIF proteins. At the base of the metazoan IF proteins, the ctenophoran lamins from *Mnemiopsis leidyi* and *Pleurobrachia pileus* are found, which is consistent with a recent study showing Ctenophora to be sister to all other animals^45^. The two lamins from the Porifera *Amphimedon queenslandica* either group to the ctenophoran lamins or at the base of the nematocilins, albeit with low support in all topologies. The single placozoan lamin from *Trichoplax adhaerens* groups out*s*ide cnidarians and bilaterians, or as sister to the cnidarians. The inconsistent grouping of the poriferan and placozoan lamins is due to the low number of sequences, but also reflects the general difficulties to resolve the phylogeny of the basal metazoan lineages^45^. Before the separation of the Cnidaria and Bilateria, the lamin gene got duplicated (Fig. 3), and the duplicate evolved into the nematocilin gene. Nematocilins are present in all eumetazoans except Ecdysozoa and Platyhelminthes. Fish and mammalian nematocilins independently lost the α-helical rod domain. The strong conservation of the nematocilins throughout eumetazoans points to an important and conserved function beyond stabilizing cnidocils in nematocytes, where nematocilin has been located in hydrozoans^20^. The bilaterian lamins group sister to the cytoplasmic mIF proteins implying that the cytoplasmic mIF proteins were derived from a bilaterian lamin and not from nematocilin. While the protostomian cytoplasmic IF proteins retained similar domain architectures to the lamins, the ancestor of the deuterostomes already reduced the cytoplasmic mIF to the α-helical rod domain. In vertebrates, the lamins were duplicated twice, resulting in doublets of a doublet in accordance with the known two whole genome duplications events^46^ (Fig. 3). The lamin A/C and lamin LIII (lamin D) subtypes, and the lamin B1 and lamin B2 subtypes group together. Some vertebrate subbranches subsequently lost certain subtypes. For example, *Callorhynchus* lost the lamin A/C and humans lost the lamin LIII subtype. The anole lizard *Anolis carolinensis* contains the lamin A/C and lamin B1 subtypes, and in addition a further very divergent lamin, which groups outside all vertebrate lamins in the phylogenetic trees. From a phylogenetic point of view, the invertebrate and non-metazoan lamins should not be classified as B-type lamins, but as lamins sharing similarity to all vertebrate lamins, as has already been pointed out^40^,^44^. Because the vertebrate lamin subtypes clearly resemble the two vertebrate whole-genome duplications, previously and repeatedly published conclusions, that B-type lamins appeared before A-type lamins, do not hold. Similarly, vertebrate lamins are not more similar to invertebrate cytoplasmatic IF proteins than they are to vertebrate cytoplasmic IFs.

## Discussion

As lamins have been identified in many major eukaryotic kingdoms the question remains when lamins appeared in eukaryotic evolution. While there is agreement for the monophyly of most eukaryotic kingdoms, there is as yet no consensus for placing the root (Fig. 3). The monophyly of the Amorphea has been shown in many analyses ruling out a proposed origin within this clade^47^,^48^. The photosynthetic-nonphotosynthetic root^49^ was based on rare amino acid substitutions and is not only strongly rejected by the other studies but also by evidence of horizontal transfer of plastids across eukaryotes^50^. Rooting the tree between Euglenozoa and all other eukaryotes (= neokaryotes) was based on a few characters like the presence of the Tom40 protein^51^ and contradicts almost all other studies. An early placement of the root between Amorphea and all other eukaryotes (unikont – bikont split^52^) was based on a single rare gene fusion, whose monophyletic character is now obsolete, but support for this root has recently been found in a multi-gene analysis of mitochondrial proteins of α-proteo-bacterial ancestry^53^. The most recent multi-gene study, also based on mitochondrial proteins, placed the root between Neozoa and Excavata. Whichever root might turn out to be correct, the current data suggest a lamin origin either in the last common ancestor of all eukaryotes or at the origin of the Neozoa (Fig. 3).

Alternatively, the presence of lamin homologs in just a single sub-branch of the stramenopiles and only a single rhizarian (note, that genome assembly data is only available for two rhizarian species so far) might indicate their origin by lateral gene transfer. In the case of the stramenopiles sub-branch, the lateral gene transfer must have happened to the ancestor of the oomycetes, and already from the ancient Amorphea as the oomycete lamins group outside all amorphean lamins. Similarly, it could be argued that the amoebozoan lamins were derived from lateral gene transfer, because lamins are not present in apusozoans and fungi, which are closer related to metazoans than amoebae, and because lamins are not present in all amoebae (e.g. not in Entamoeba and Acanthamoeba). However, several facts strongly argue against the origin of lamins by lateral gene transfer. First, the lamin gene tree is in agreement with the most commonly accepted species tree. Second, lateral gene transfer must have happened several times independently, and all transferred lamins must have subsequently evolved so that they group now as if they had evolved according to the species tree. Third, gene loss and gene gain, and also domain loss and domain gain, are very common and have happened all the time during eukaryotic evolution. The entire history of the metazoan lamins and intermediate filament proteins is characterized by numerous gene duplication and independent gene loss events (Fig. 3). Similar patterns have been found for many other cytoskeletal proteins^29^,^34^,^36^,^54^. Therefore, it seems more parsimonious that a lamin was present in the last eukaryotic ancestor, and that the lamins were lost in many early eukaryotic branches or diverged so that they cannot be identified by sequence similarity searches anymore.

Currently, a generally agreed definition of an “intermediate filament protein” does not exist. In the first three decades of IF protein research, intermediate filament proteins had been described by their thickness (about 10 nm) and by their ability to form unbranched fibers extending from the cell nucleus to the plasma membrane. All IF proteins known at that time showed cross-reactivity with the same monoclonal antibody^55^. However, these descriptions have been challenged by the discovery of network-forming filaments such as the lens-specific proteins filensin and phakinin^56^. In addition, intermediate filament-like proteins have been identified whose sequence similarity to the old, canonical IFs is not readily visible. These proteins include the nestins, which are currently restricted to vertebrates, the euglenozoan-specific articulins, the alveolins, which are restricted to alveolates, and the tetrins and epiplasmins currently restricted to Ciliophora. Articulins do not even contain coiled-coil regions. Thus, how did these proteins evolve? Phakinins show high similarity to type I to type IV IFs and might form a subgroup of one of these types. The N-termini of filensins and nestins can be aligned to the other metazoan IFs^18^, they have the same number of heptads as the type I to type IV IFs, and show gene structure homology to the type IV IFs, as has been noted earlier^57^. Phakinins, filensins and nestins thus clearly evolved from one of the canonical type I to type IV IFs (Fig. 3). The alveolins, articulins, tetrins, and epiplasmins do not show any sequence similarity to the metazoan IFs. Thus, they do not contain the “consensus sequences” and not at all a similarly organized α-helical rod domain, that characterize the metazoan IFs^58^. Alveolins, tetrins, and epiplasmins could have evolved from any ancient coiled-coil containing protein. Because lamin homologs with conserved domain architectures and highly similar coiled-coil regions have been identified in oomycetes and rhizarians, convergent evolution seems more likely than a shared ancestry of the alveolate and euglenozoan intermediate filament-like and the metazoan intermediate filament proteins. Recently, an intermediate filament forming protein called crescentin has been identified in the bacterium *Caulobacter crescentus*^59^. Crescentin homologs are also present in *Brevundimonas*, *Microvirga*, and *Methylobacterium* bacteria. They contain long coiled-coil regions, which however do not show more similarity to the metazoan intermediate filament proteins than to other coiled-coil containing cytoskeletal proteins. All this rather indicates an origin by convergent evolution.

Until now lamins and the canonical IF proteins have been restricted to Metazoa and Amoebozoa. Nuclear lamina have experimentally been shown for many protists, but protozoan sequences never showed sequence homology to metazoan IF proteins beyond coiled-coil regions. Although dozens of protozoan genome sequences have been available for years (e.g. the *Phytophthora ramorum* genome has been published eight years ago^60^), sequence similarity searches never revealed any mIF protein homologs. However, here I found lamin homologs in many oomycetes, a Rhizaria species, in Ichthyosporea and choanoflagellates. Although I tried hard to detect further homologs in the branches where mIF proteins have not been identified yet, there still might be lamins and mIF proteins not detectable with current methods. This might also be true for further types of mIF proteins in metazoans like the nematocilins, which have just recently been identified. The presented data on mIF proteins provide a foundation for experimental research of protozoan nuclear lamina, and for studying further aspects of nuclear envelope evolution.

## Methods

### Identification and annotation of the lamin genes

Lamin and cytoplasmic mIF genes were identified in iterated TBLASTN searches in the completed or almost completed genomes of 305 species starting with the protein sequence of human lamin-A. The genome sequences covering these search hits, were submitted to the de-novo gene predictions software AUGUSTUS^61^ to obtain preliminary protein sequences. A considerable limit of gene prediction software is the lack of appropriate species-specific feature sets. For example, feature sets for stramenopiles and choanoflagellates are not available. Gene prediction errors become apparent when adding the predicted sequences to the multiple sequence alignment of the already annotated and corrected lamins and cytoplasmic mIFs (see below). Therefore, gene predictions were corrected, when necessary and where possible, through manual analysis of the respective genomic regions. In some cases, available EST (Expressed Sequence Tag) data helped resolving regions, for which comparative genomic data was not available. In particular, EST data was used to validate the absence of important sequence motifs. For example, the C-termini of the *Helobdella robusta* lamins, which miss the CaaX motif, are supported by many EST clones. Gene prediction is highly facilitated if homologs of closely related species have already been obtained. In such cases, I used the gene reconstruction software WebScipio with default cross-species search parameters^62^,^63^. By this approach, better gene predictions are obtained and even gene duplicates correctly determined. However, this requires a certain sequence similarity and, thus, does not ensure that all lamin and mIF homologs are identified. Therefore, the respective species were also analysed by TBLASTN searches. For example, the *Anolis carolinensis* lamin-A and lamin-B1 could correctly be reconstructed by WebScipio, while the divergent lamin-E would have been missed without TBLASTN searches.

Sequences were termed “Partials”, if up to 5% of the supposed full-length sequences were missing due to gaps in the genome assemblies. Sequences of which more than 5% were missing due to genome assembly gaps or incomplete EST data but which are otherwise unambiguous orthologs or paralogs were termed “Fragments”. These “Partials” and “Fragments” were removed from the alignments used in the phylogenetic analyses, but are important to denote the presence of the respective protein homologs in the respective species.

IF genes were termed pseudogenes if they consisted of single pseudo-coding exons that contained deletions and insertions (which would lead to frame shifts in the translations), in-frame stop codons, and/or missed considerable parts of a “normal” full-length lamin. While this procedure was reliable for annotating for example the mouse lamin pseudogene, presuming that the respective genome assembly is almost complete and does not contain any sequencing and assembly errors, annotating pseudogenes in other species was more difficult. For example, several of the low-coverage vertebrate and insect genome assemblies are of low quality and it would not be surprising to observe sequencing and assembly errors within coding regions. In these cases, I did not annotate the respective IF genes as pseudogenes.

The *Branchiostoma floridae* lamin and some of the cytoplasmic IF genes encode alternative splice variants. The different splice forms were not considered independently in the analysis but in all cases the same splice forms were taken for homologous IFs. All sequences are available at CyMoBase ( www.cymobase.org)^64^. CyMoBase offers search and filter functions for the data, and provides many additional information such as GenBank ID’s, gene structure representations, previously used (alternative) names, primary publications to previous sequence reports, domain predictions, and statistical analyses Also, the genome sequencing centers are referenced for each species

### Generating the multiple sequence alignment

The lamin/mIF sequence alignment in its current stage was created over years of assembling lamin sequences. The initial alignment was created based on a few full-length cDNA sequences obtained from GenBank. Automatic alignment of divergent or gap-containing sequences is error prone. Therefore, substantial efforts have been undertaken to manually improve the alignment. To not rebuild the main alignment with every added sequence, newly predicted sequences were first aligned separately to their supposed closest relative using ClustalW^65^. Subsequently, these “aligned” sequences were added to the main alignment, and the alignment manually improved where possible. The newly predicted sequences often contain mispredicted regions (see above), which become obvious through inspecting the alignment. Together with manually adjusting the alignment, wrongly predicted sequences were removed and gap regions filled. Still, many gaps remained in sequences derived from low-coverage genomes. In these cases, alignment gaps were added as place holders while maintaining the integrity of exons preceding and following the gaps. Automatic alignment software is not aware of sequence gaps due to genome assembly gaps, and thus often spreads the sequence over the gap region. The alignment of the lamins/mIFs can be obtained from CyMoBase ( www.cymobase.org)^64^ and as Supplementary Data S1.

### Preparation of the datasets for the phylogenetic analyses

The dataset contained in total 892 lamins and cytoplasmic mIFs. To generate datasets for phylogenetic analyses, “pseudogenes” and sequences with gaps resulting from genome assembly gaps were removed from the multiple sequence alignment. The resulting alignment of 729 lamins and cytoplasmic mIFs was treated with CD-Hit v.4.5.4 (similarity threshold of 90%)^66^ and gblocks v.0.91b^67^ to generate datasets with less redundancy and smaller blocks. The gblocks parameters were as follows: A) The minimum number of sequences for a conserved position and the minimum of sequences for a flank position were set to the minimum (e.g. half the number of sequences plus one). B) The maximum number of contiguous nonconserved positions was set to 8 and the minimum length of a block was set to 5. C) The parameter for the allowed gap position was set to ‘with half’ meaning that only positions within 50% or more of the sequences having a gap are treated as gap positions. In addition, datasets excluding the amoebozoan lamins, both the *Oikopleura dioica* and a set of Platyhelminth lamins, or all these lamins were generated to investigate branch attraction effects between these sequences. Finally, datasets were created, in which highly variable regions were removed manually.

### Computing and visualising phylogenetic trees

Phylogenetic trees were generated using the Neighbour Joining (1) and the Maximum likelihood (2) method. 1. Unrooted trees with 1,000 bootstrap replicates were calculated with ClustalW v.2.0.10^65^. Trees were corrected for multiple substitutions. 2. FastTree v.2^68^ was used to perform Maximum likelihood (ML) analyses with estimated proportion of invariable sites and bootstrapping (1,000 replicates). FastTree offers the JTT^69^ and WAG^70^ amino acid substitution models. The more appropriate model was determined by using ProtTest v.3.2^71^ calculating the tree topology with the BioNJ algorithm and optimizing both the branch lengths and the model of protein evolution simultaneously. According to the Akaike Information Criterion (AIC), the JTT+Γ was determined to be the best model. Phylogenetic trees were visualized with FigTree ( http://tree.bio.ed.ac.uk/software/figtree/).

## Additional Information

**How to cite this article**: Kollmar, M. Polyphyly of nuclear lamin genes indicates an early eukaryotic origin of the metazoan-type intermediate filament proteins. *Sci. Rep.* **5**, 10652; doi: 10.1038/srep10652 (2015).

## Supplementary Material

Supplementary Information

Supplementary Information
