# Supplementary material for: Polyphyly of nuclear lamin genes indicates an early eukaryotic origin of the metazoan-type intermediate filament proteins
Source: Sci Rep. 2015 May 29;5:10652. doi: 10.1038/srep10652 (PMC4448529; doi:10.1038/srep10652)
Supplement: Supplementary Information [file srep10652-s2.pdf]

Supplementary Information

Polyphyly of nuclear lamin genes indicates an early eukaryotic origin of the metazoan-type intermediate filament proteins

Martin Kollmar

Group Systems Biology of Motor Proteins, Department of NMR-based Structural Biology, Max-Planck-Institute for Biophysical Chemistry, Göttingen, Germany

mako@nmr.mpibpc.mpg.de

Table S1. EST/cDNA/TSA databases contain many lamin and IF gene hits that could be shown to be contaminations in the DNA libraries.

genbank ID(s)	species given in genbank entry	ORF most closely related or identical to
GR355753, GR347622	Avena barbata, Viridiplantae	deuterostomian cytoplasmic IF
EH036144	Symbiodinium sp. C3, Alveolata	Acropora digitifera lamin, Cnidaria
HO505420, HO567473, HO493982, HO565171	Nitella hyalina, Viridiplantae	Adineta vaga lamin, Lophotrochozoa
GT044443	Festuca arundinacea, Viridiplantae	Aphanomyces euteiches lamin, oomycete
GAAW01020108	Humulus lupulus, Viridiplantae	Tetranychus urticae lamin, arthropod
GACH01085389	Chromolaena odorata, Viridiplantae	Heterodera glycines lamin, nematod
GADD01017671	Pyropia haitanensis, Rhodophyta	mammalian keratin81
CV445723	Boophilus microplus, arthropod	mammalian keratin17
GT041775, GT045861	Festuca arundinacea, Viridiplantae	groups outside metazoan and amoebozoan lamin genes

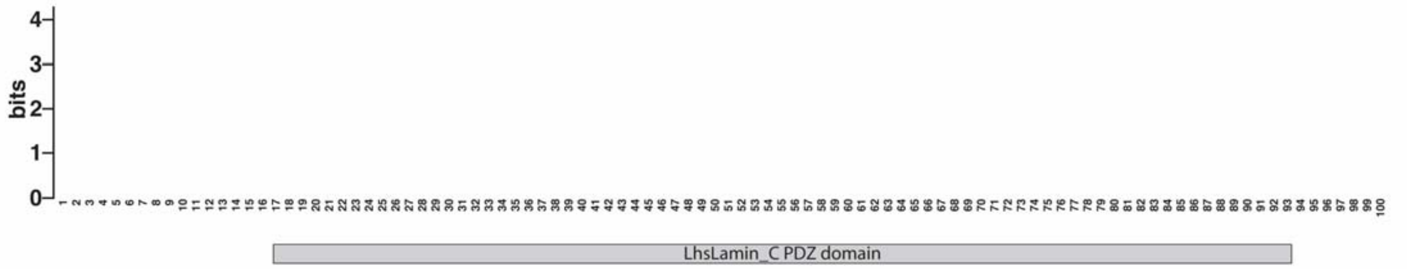


```
                                  10        20        30        40        50        60        70        80        90       100
                         ....|....|....|....|....|....|....|....|....|....|....|....|....|....|....|....|....|....|....|....|
HsLamin_A         1      ----------------------------------------------------------------------------------------------------
AlsiLamin_A       1      ----------------------------------------------------------------------------------------------------
HsLamin_B1        1      ----------------------------------------------------------------------------------------------------
HsLamin_B2        1      ----------------------------------------------------------------------------------------------------
AocLamin_E        1      ----------------------------------------------------------------------------------------------------
BrLamin_LIII      1      ----------------------------------------------------------------------------------------------------
XlLamin_LIIIb     1      ----------------------------------------------------------------------------------------------------
PtmLamin_C        1      ----------------------------------------------------------------------------------------------------
SmLamin           1      ----------------------------------------------------------------------------------------------------
LhsLamin_C        1      MVVSWRPRSITVDRGTRGFGLSLIYRGLDKYEQKDTGIFVARVVKDGQAEKYGVRENDKIVSINAKTPRNVDDAVNVIKSAGNQIKLVVLREEDVPDNDD
DmLamin_A         1      ----------------------------------------------------------------------------------------------------
AngLamin_A        1      ----------------------------------------------------------------------------------------------------
CeLamin           1      ----------------------------------------------------------------------------------------------------
MnlLamin          1      ----------------------------------------------------------------------------------------------------
AcdLamin          1      ----------------------------------------------------------------------------------------------------
CoLamin           1      ----------------------------------------------------------------------------------------------------
CrtLamin          1      ----------------------------------------------------------------------------------------------------
Phr_aLamin        1      ----------------------------------------------------------------------------------------------------
DdLamin           1      ----------------------------------------------------------------------------------------------------

HsVimentin        1      ----------------------------------------------------------------------------------------------------
HsNF-L            1      ----------------------------------------------------------------------------------------------------
MdPeripherin      1      ----------------------------------------------------------------------------------------------------
HsKeratin18       1      ----------------------------------------------------------------------------------------------------
XtNem             1      ----------------------------------------------------------------------------------------------------
NvNem             1      ----------------------------------------------------------------------------------------------------
SckNem_A          1      ----------------------------------------------------------------------------------------------------
CeIF_A            1      ----------------------------------------------------------------------------------------------------
LpIF_A            1      ----------------------------------------------------------------------------------------------------
```

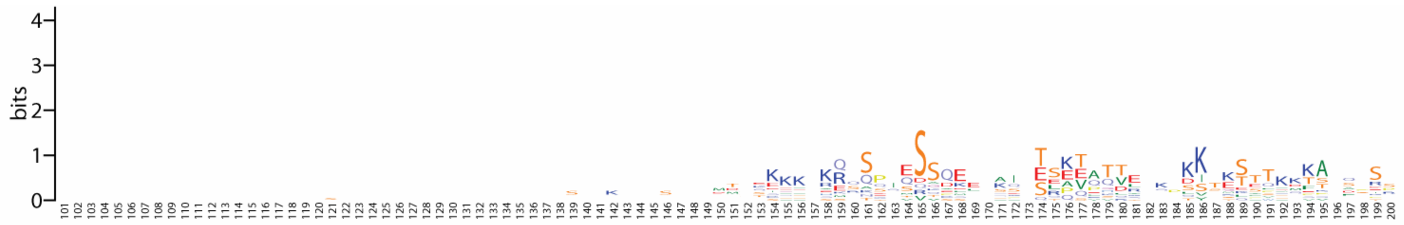

```
                        110       120       130       140       150       160       170       180       190       200
                ....|....|....|....|....|....|....|....|....|....|....|....|....|....|....|....|....|....|....|....|
HsLamin_A     1 ----------------------------------------------------------------------------------------------------
AlsiLamin_A   1 ----------------------------------------------------------------------------------------------------
HsLamin_B1    1 ----------------------------------------------------------------------------------------------------
HsLamin_B2    1 ----------------------------------------------------------------------------------------------------
AocLamin_E    1 ----------------------------------------------------------------------------------------------------
BrLamin_LIII  1 ----------------------------------------------------------------------------------------------------
XlLamin_LIIIb 1 ----------------------------------------------------------------------------------------------------
PtmLamin_C    1 ----------------------------------------------------------------------------------------------------
SmLamin       1 ----------------------------------------------------------------------------------------------------
LhsLamin_C  101 PNDHSSIASHDWLHGHPISRSGSARSFNTTYGGGNASPSPLPSKSPGPQRTSQSDFLRQQAEYQRQQQQQKQLEEEEEHRRKQMLYEEEELRRKQMHYEE
DmLamin_A     1 ----------------------------------------------------------------------------------------------------
AngLamin_A    1 ----------------------------------------------------------------------------------------------------
CeLamin       1 ----------------------------------------------------------------------------------------------------
MnlLamin      1 ----------MPKKKTPAKSSGKQKAEAPPAEDKSPPPSKAKDLESSEAQEKEIVTTKSFSSTTSTRKGTYSQSREEYQVLPRFKIPRLSWDDVAIDLDD
AcdLamin      1 ----------------------------------------------------------------------------------------------------
CoLamin       1 ----------------------------------------------------------------------------------------------------
CrtLamin      1 ----------------------------------------------------------------------------------------------------
Phr_aLamin    1 ----------------------------------------------------------------------------------------------------
DdLamin       1 -------------------------------------------------MDMSKKKSKRASPIESSQEEIAISTSKTATTE-KPKKTKTTTKKKASQPS-

HsVimentin    1 --------------------------------------------------------------------------MSTRSVSSSSYRRMFGGPGTASRPSS
HsNF-L        1 ---------------------------------------------------------------------MSSFSYEPYYST--SYKRRYV----------
MdPeripherin  1 --------------------------------------------------------------------MSHL-SGLRTSSTSTSYRRTFGAPASLS----
HsKeratin18   1 ----------------------------------------------------------------------MSFTTRS-TFST-NYRSLGSVQAPSYGARP
XtNem         1 ----------------------------------------------------------------------------------------------------
NvNem         1 ----------------------------------------------------------------------------------------------------
SckNem_A      1 ----------------------------------------------------------------------------------------------------
CeIF_A        1 ----------------------------------------------------------------------------------------------------
LpIF_A        1 ---------------------------------------------------------------------------MSVTQKKTEISTTTTYEGESRPSSG
```

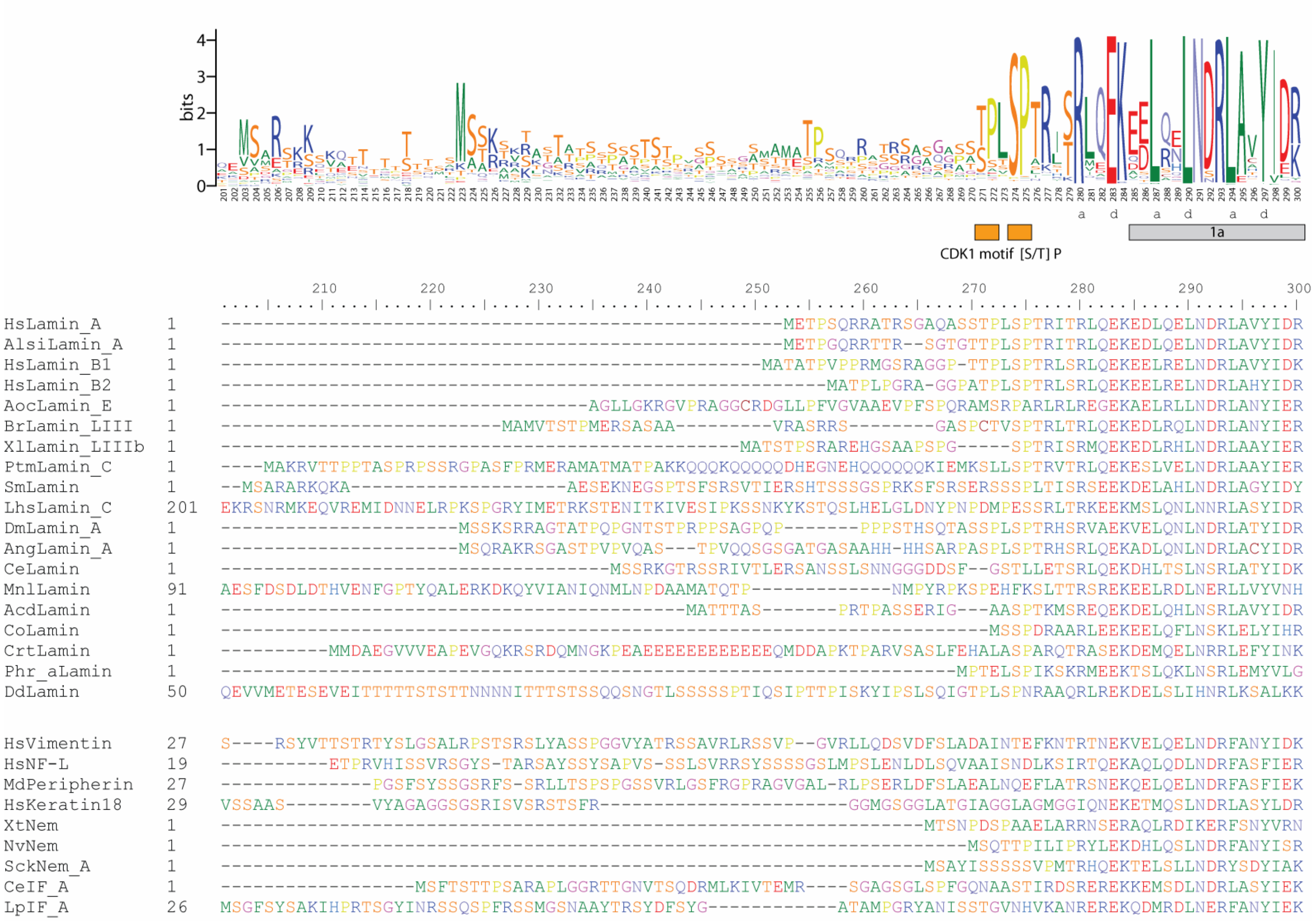

bits
a d a d a d
1a
CDK1 motif [S/T] P
210 220 230 240 250 260 270 280 290 300
HsLamin_A 1 ---METPSQRRATRSGAQASSTPLSPTRITRLQEKEDLQELNDRLAVYIDR
AlsiLamin_A 1 ---METPGQRRTTR--SGTGTTPLSPTRITRLQEKEDLQELNDRLAVYIDR
HsLamin_B1 1 ---MATATPVPPRMGSRAGGP-TTPLSPTRLSRLQEKEELRELNDRLAVYIDK
HsLamin_B2 1 ---MATPLPGRA-GGPATPLSPTRLSRLQEKEELRELNDRLAHYIDR
AocLamin_E 1 ----------------------------------AGLLGKRGVPRAGGCRDGLLPFVGVAAEVPFSPQRAMSRPARLRLREGEKAELRLLNDRLANYIER
BrLamin_LIII 1 --------------------------MAMVTSTPMERSASAA---------VRASRRS--------GASPCTVSPTRLTRLQEKEDLRQLNDRLANYIER
XlLamin_LIIIb 1 --MATSTPSRAREHGSAAPSPG-----SPTRISRMQEKEDLRHLNDRLAAYIER
PtmLamin_C 1 ----MAKRVTTPPTASPRPSSRGPASFPRMERAMATMATPAKKQQQKQQQQQDHEGNEHQQQQQQKIEMKSLLSPTRVTRLQEKESLVELNDRLAAYIER
SmLamin 1 --MSARARKQKA--------------------AESEKNEGSPTSFSRSVTIERSHTSSSGSPRKSFSRSERSSSPLTISRSEEKDELAHLNDRLAGYIDY
LhsLamin_C 201 EKRSNRMKEQVREMIDNNELRPKSPGRYIMETRKSTENITKIVESIPKSSNKYKSTQSLHELGLDNYPNPDMPESSRLTRKEEKMSLQNLNNRLASYIDR
DmLamin_A 1 -----------------------MSSKSRRAGTATPQPGNTSTPRPPSAGPQP-------PPPSTHSQTASSPLSPTRHSRVAEKVELQNLNDRLATYIDR
AngLamin_A 1 -----------------------MSQRAKRSGASTPVPVQAS---TPVQQSGSGATGASAAHH-HHSARPASPLSPTRHSRLQEKADLQNLNDRLACYIDR
CeLamin 1 ------------------------------------MSSRKGTRSSRIVTLERSANSSLSNNGGGDDSF--GSTLLETSRLQEKDHLTSLNSRLATYIDK
MnlLamin 91 AESFDSDLDTHVENFGPTYQALERKDKQYVIANIQNMLNPDAAMATQTP-------------NMPYRPKSPEHFKSLTTRSREKEELRDLNERLLVYVNH
AcdLamin 1 ---MATTTAS-------PRTPASSERIG---AASPTKMSREQEKDELQHLNSRLAVYIDR
CoLamin 1 --MSSPDRAARLEEKEELQFLNSKLELYIHR
CrtLamin 1 ----------MMDAEGVVVEAPEVGQKRSRDQMNGKPEAEEEEEEEEEEEEQMDDAPKTPARVSASLFEHALASPARQTRASEKDEMQELNRRLEFYINK
Phr_aLamin 1 ---MPTELSPIKSKRMEEKTSLQKLNSRLEMYVLG
DdLamin 50 QEVVMETESEVEITTTTTSTSTTNNNNITTTSTSSQQSNGTLSSSSSPTIQSIPTTPISKYIPSLSQIGTPLSPNRAAQRLREKDELSLIHNRLKSALKK
HsVimentin 27 S----RSYVTTSTRTYSLGSALRPSTSRSLYASSPGGVYATRSSAVRLRSSVP--GVRLLQDSVDFSLADAINTEFKNTRTNEKVELQELNDRFANYIDK
HsNF-L 19 ----------ETPRVHISSVRSGYS-TARSAYSSYSAPVS-SSLSVRRSYSSSSGSLMPSLENLDLSQVAAISNDLKSIRTQEKAQLQDLNDRFASFIER
MdPeripherin 27 --------------PGSFSYSSGSRFS-SRLLTSPSPGSSVRLGSFRGPRAGVGAL-RLPSERLDFSLAEALNQEFLATRSNEKQELQELNDRFASFIEK
HsKeratin18 29 VSSAAS--------VYAGAGGSGSRISVSRSTSFR-----------------------GGMGSGGLATGIAGGLAGMGGIQNEKETMQSLNDRLASYLDR
XtNem 1 ---MTSNPDSPAAELARRNSERAQLRDIKERFSNYVRN
NvNem 1 ---MSQTTPILIPRYLEKDHLQSLNDRFANYISR
SckNem_A 1 ---MSAYISSSSSVPMTRHQEKTELSLLNDRYSDYIAK
CeIF_A 1 ------------------MSFTSTTPSARAPLGGRTTGNVTSQDRMLKIVTEMR----SGAGSGLSPFGQNAASTIRDSREREKKEMSDLNDRLASYIEK
LpIF_A 26 MSGFSYSAKIHPRTSGYINRSSQSPFRSSMGSNAAYTRSYDFSYG------------ATAMPGRYANISSTGVNHVKANREREKQDMRDLNERFANYIEK

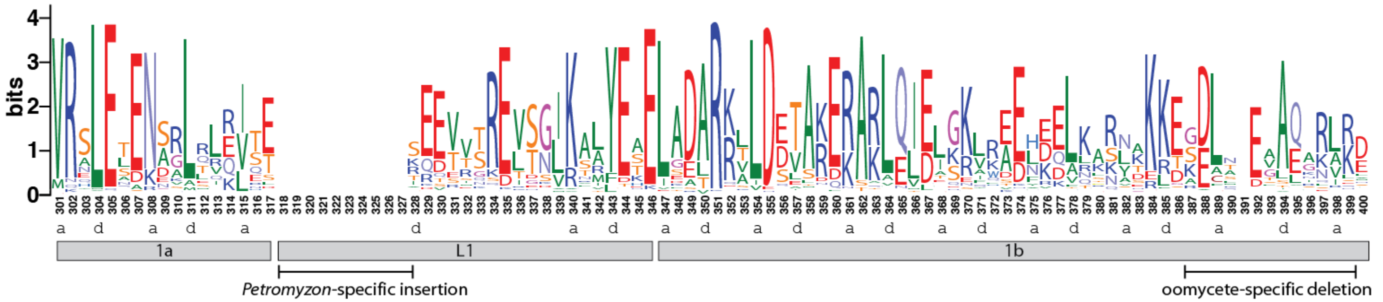


```
                     310       320       330       340       350       360       370       380       390       400
                .........|.........|.........|.........|.........|.........|.........|.........|.........|.........|
HsLamin_A     49  VRSLETENAGLRLRITE----------SEEVVSREVSGIKAAYEAELGDARKTLDSVAKERARLQLELSKVREEFKELKARNTKKEGDLI--AAQARLKD
AlsiLamin_A   47  VRSLESENAGLRLRITE----------SEEVVSREVSGIKVAYESELADARKTLDSVAKERARLQLELSKVREEHKELKARNAKKESDLL--SAQSRLKD
HsLamin_B1    50  VRSLETENSALQLQVTE----------REEVRGRELTGLKALYETELADARRALDDTARERAKLQIELGKCKAEHDQLLLNYAKKESDLN--GAQIKLRE
HsLamin_B2    44  VRALELENDRLLLKISE----------KEEVTTREVSGIKALYESELADARRVLDETARERARLQIEIGKLRAELDEVNKSAKKREGELT--VAQGRVKD
AocLamin_E    67  VQALELQNGRLLLRVSHEE--------NEEQRQKQPGEARGLHGAHQAR-REGHDSAANR--LLREKAATLRAQIEQTESSLKKKEGECV--WWRSRAQE
BrLamin_LIII  58  VRQLEADKSSMQILLEE----------KEESSLREVGNVRRLYESELADARKSLDATANERARLQIEFSQLQEDHRKLAARNNKKECELT--AAVDRWRN
XlLamin_LIIIb 48  VRSLEADKSLLKIQLEE----------REEVSSREVTNLRQLYETELADARKLLDQTANERARLQVELGKVREEHRQLQARNNKKETDLC--LVQNQLRD
PtmLamin_C    97  VRSLEVDKARLLARVSMWEEEEEEQRQKSERQREATGGLRAAYDAELAEVRRALDDALGAKAALEIQLRLAQVERDEGRSRCSKYEQEWN--VALGRIRD
SmLamin       79  VRKLELDKQKLTRRIQT----------VTEERMSKVEEARKTYEDEITALRNLVDDLAKQKSKAELDSKQMRDELNDIKMKANKRDQENR--NLQRKIEN
LhsLamin_C    301 VRQLQNENNKLSHQIKT----------VEEYQSKELCNVKDLYDKQTEELKEALDTMNKQYNQLKVGAEGLLEENQDLKDKLRKKENDYK--NSMNHVSS
DmLamin_A     72  VRNLETENSRLTIEVQT----------TRDTVTRETTNIKNIFEAELLETRRLLDDTARDRARAEIDIKRLWEENEELKNKLDKKTKECT--TAEGNVRM
AngLamin_A    75  VRYLEQENSRLTMEVRT----------SQDTTTREVSNIKSMYEHELSDARKLLDDTSREKARLEIDGKRILEENDELKKRLERKTKEAA--DADRNARA
CeLamin       63  VRQLEQENNRLQVQIRD----------IEVVEKKEKSNLADRFEAEKARLRRALDSAQDELAKYRIEYDAAKVEVKKLKPQVEKLERELA--GAEEQALH
MnlLamin      178 MRSLKEIEETEEHIQVD------------GELTKIQESLKDLFHTEIEEARRLLDETANQKAALELEAGRHLDTIEQLKKELAEKDEEIE--RLKKLIGE
AcdLamin      48  VNALEESNNKLTAEITL----------TKSRTRTEVESVKNSFESELADARRLLDETAKEKAREQMENRKNASVADEFKKKFEKEVAAHK--KTEDELKA
CoLamin       30  RQELQTLVKTLESELER----------ERNDAQQRLEIAHRQYEATLTETRHALDKAASEASHASSSLVQVNAELAATKERARTESSAAD--ALRSRYEA
CrtLamin      91  QREREARRSTEERELMT----------AKQVVSREIDRARAEYQSQLAEVRKQRDQHVASKTHLQTTIARLQQNVYDLEGRLKQEQAEHK--ELESRRDQ
Phr_aLamin    33  VNELEGAKHAAERELET----------IKQRMQQDMESVRSRLTKELEETRKKLDDEMDQNARLQTLEQEQHKELVKLRAQQTELG-------------E
DdLamin       150 LESAETELEKKNQEYEE----------LDQKHTATIKQLKQRSDQVEKQLIEEQNQNSDLTSNRNILENELKSKESVWKKEKDEILLKFQ-ESINKLNQE

HsVimentin    121 VRFLEQQNKILLAELE-------------QLKGQGKSRLGDLYEEEMRELRRQVDQLTNDKARVEVERDNLAEDI------------------MRLREKL
HsNF-L        108 VHELEQQNKVLEAELLVL-----------RQKHSEPSRFRALYEQEIRDLRLAAEDATNEKQALQGEREGLEETL------------------RNLQARY
MdPeripherin  112 VRFLEQQNAALRGELSQV-------------RGQEPARADQLCQQELRELRRELELLGRDRDRVQVERDGLAEDL------------------AALKQRL
HsKeratin18   98  VRSLETENRRLESKIREH-----------LEKKGPQVRDWSHYFKIIEDLRAQIFANTVDNARIVLQIDNARLAA------------------DDFRVKY
XtNem         36  VRVLRNQLTQGEGFT------------AAPRLEQEVLGIRANYEQEIQSLRAALEKYRELAQGGNHRETPT---APEYHSRLLELSRDLI--KKDQEIRH
NvNem         32  VRQMREHNGRNETINFIN---------TTKILEEEILALKAMYERQLEELRSKLDDVARDRTQQQMANAKNSALVAELQDKLGEETSNRK--KVENALAD
SckNem_A      36  VRKLQQEANQDDSSALLN---------SIRNLEEEINSIKALYDNELDKLRRELEGVTSEKQRLELFAKKNNGLATELQDRLSVEQRQNQ--ALIGELGN
CeIF_A        79  VRFLEAQNRKLAADLDA----------LRSKWGKDTHNIRNMYEGELVDAQKLIDETNKQRKDMEGQLKKMQDELAEMRRKLEDATKGRE--QDRAKIDA
LpIF_A        114 VRFLEAQNKKLAGELEE----------LKSKWGKETSAIKEMYETELEEARKLIDATNKEKITLDVRVTELIDQLERQQKDLEESRTYHQ--IDQEQIAR
```

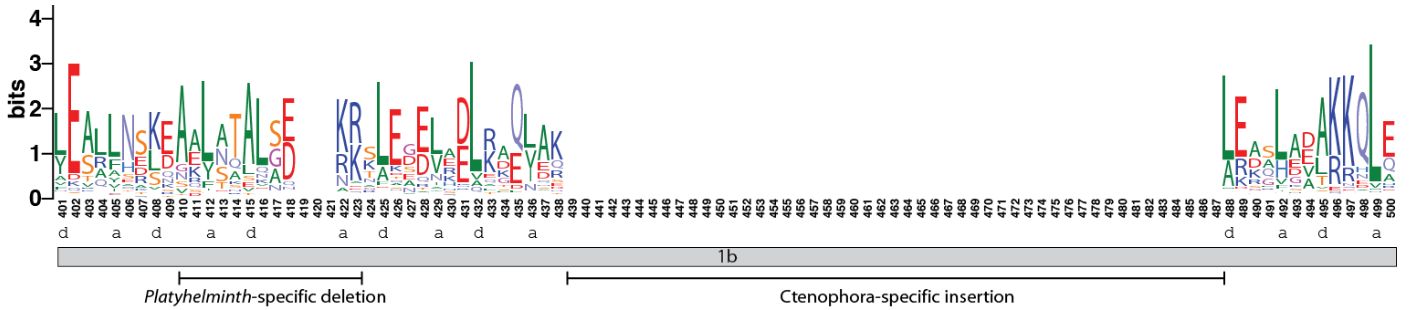


```
                                    410       420       430       440       450       460       470       480       490       500
HsLamin_A        137   LEALLNSKEAALSTALSE---KRTLEGELHDLRGQVAK-------------------------------------------------LEAALGEAKKQLQ
AlsiLamin_A      135   LEALLNSKEAALTTALGE---KRNLENEVRELRAQVAK-------------------------------------------------LEASLNEAKKQLQ
HsLamin_B1       138   YEAALNSKDAALATALGD---KKSLEGDLEDLKDQIAQ-------------------------------------------------LEASLAAAKKQLA
HsLamin_B2       132   LESLFHRSEVELAAALSD---KRGLESDVAELRAQLAK-------------------------------------------------AEDGHAVAKKQLE
AocLamin_E       154   LEGLLRGREGDLATALAR---KRDLEAEVDALRTRLAQ-------------------------------------------------SEESQAVAKRQLE
BrLamin_LIII     146   LEVALNAKEADFANLLST---NRRLETEISELKTQVPA-------------------------------------------------LEAALQKLKTQLN
XlLamin_LIIIb    136   FESKLNTKEAELATTLSG---KRGLEEQLQEQRAQITS-------------------------------------------------LESSLRDTTKQLH
PtmLamin_C       195   MEVQLCAKEASLSTASGD---KEYLETEVDDLRNQLNK-------------------------------------------------LEGVLVSSKQQLE
SmLamin          167   LERELSKYK--------------QDHDAYQPLLSDYHV-------------------------------------------------LEKRFEEMKRDLE
LhsLamin_C       389   LEDQIRSLTNKLSEEVTE---RKKIADELQDVLPELDS-------------------------------------------------LRDRLADAKRHLD
DmLamin_A        160   YESRANELNNKYNQANAD---RKKLNEDLNEALKELER-------------------------------------------------LRKQFEETRKNLE
AngLamin_A       163   QEARANELSTKYHSLVSE---HKKAKDDQKELEKELAK-------------------------------------------------LRKQLDALRKNLE
CeLamin          151   AQSIADQSQAKQKTLQAR---NDKLVVENDDLKKQNIT-------------------------------------------------LRDTVEGLKKAVE
MnlLamin         264   LETKLANALGDIKEAKAD---NKRLHKENDELRKQLADEQNNRKSSEKEIVRLERVISNYESDISKLKQQLKEYADKEKAMKNELKNLRKNLEDLKDELD
AcdLamin         136   SHRKLSEKEAQLSVVNQE---ARSLQSVLEELRKECQE-------------------------------------------------LKDSLESAKYALE
CoLamin          118   LQLAKHELDGRLAVSEIA---IKELKDNLKHADSDRKH-------------------------------------------------AEKESDALRKSSE
CrtLamin         179   LSNQLKDVNKELQNAKEL---LRSTEGDLEDQQKQNDA-------------------------------------------------LEGEIDRLRKEYE
Phr_aLamin       110   AKVLVETLRIQLDREKAN---AESAKETLSEQTTQLNS-------------------------------------------------ARRRVKELERDMR
DdLamin          239   NSLAQSQLKSEIVSKEYE---IDGLKSEINRLKDDLQY-------------------------------------------------RIREGEEKSRKLL

HsVimentin       190   QEEMLQREEAENT------------------------------------------------------------------------------LQSFRQDVD
HsNF-L           179   EEEVLSREDAEGR------------------------------------------------------------------------------LMEARKGAD
MdPeripherin     181   EEEIHKREDAENN------------------------------------------------------------------------------LVLFRKDVD
HsKeratin18      169   ETELAMRQSVEND------------------------------------------------------------------------------IHGLRKVID
XtNem            119   LQLLMAQKEAEMQGLKTA---AVTPSIQLDLAKQELKD-------------------------------------------------LHQSLCGAQERYE
NvNem            121   AHRVIAEKEAQLQDARVT---VTQHQNDHTDTRRDRDS-------------------------------------------------LQSTLTQTQQALD
SckNem_A         125   LQQLLGKKDLEINQLAGE---RDDLAKKFNDLQRDYDG-------------------------------------------------LLRDFEEVSRKLE
CeIF_A           167   LLVTLSNLEAEISLLKRR---IAQLEDEVKRIKQENQR-------------------------------------------------LLSELQRARTDLD
LpIF_A           202   QNQQLADLEGEISMLRRS---IESLEKEKMRQSNILAK-------------------------------------------------MNDEMEKMRMDLN
```

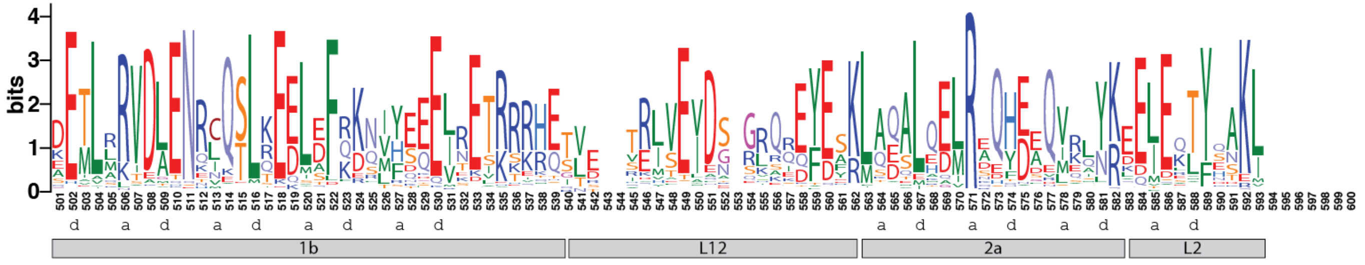


```
                          510       520       530       540       550       560       570       580       590       600
                  ....|....|....|....|....|....|....|....|....|....|....|....|....|....|....|....|....|....|....|....|
HsLamin_A     185 DEMLRRVDAENRLQTMKEELDFQKNIYSEELRETKRRHE-----TRLVEIDN-GKQREFESRLADALQELRAQHEDQVEQYKKELEKTYSAKL-------
AlsiLamin_A   183 DEMLRRVDAENRLQTLKEELEFQKNIYSEELRETKRRHE-----TRLVEIDN-GRQREFESKLSEALHDLRAQHEAQINLYKEELEKTYSAKL-------
HsLamin_B1    186 DETLLKVDLENRCQSLTEDLEFRKSMYEEEINETRRKHE-----TRLVEVDS-GRQIEYEYKLAQALHEMREQHDAQVRLYKEELEQTYHAKL-------
HsLamin_B2    180 KETLMRVDLENRCQSLQEELDFRKSVFEEEVRETRRRHE-----RRLVEVDS-SRQQEYDFKMAQALEELRSQHDEQVRLYKLELEQTYQAKL-------
AocLamin_E    202 AETLLRVDLENRNMSLREEMDFQKSLFQQEVRQ---REE----RPLVADPGPAQKEAPPPVPLSEALEDLRRQHQAQVELYREEVEQAYRAKL-------
BrLamin_LIII  194 SEMLRRVDAENQIQTLQEQLDFQKHLGEQEVREMRSRHE-----SRLLELDC-GRQKEFEGKLAEAMKQLRDEHETQIRQYKDELEKTFNAKL-------
XlLamin_LIIIb 184 DEMLWRVDLENKMQTIREQLDFQKNIHEQEVKEIKKRHD-----SRIVEIDS-GRRIEFESKLAEALQELRSDHEQQISDYKEQLEKNFSAKIHFTLDTI
PtmLamin_C    243 EETLARVDLENRMLTLQEELEFTKRIHEQEIHESRSKRE-----THVLEIDG-GQQQAFEYKLSEALTELRQQHEEQVQLYRQELDQTMRAKL-------
SmLamin       204 AETLLRTDLENKILGLKEQLDFRSRLFDEEREKLVERSL-----YIEEEVEG-RKQAEYESRLTEELRSIRDQTASELEEYKIQMEETFESKL-------
LhsLamin_C    437 EEQLKKANLENQCSRLEEDLKFKLQLLEKELMEVKTRKE-----IEITEMDS-KLQDEYEDRLQKALEELREVYDKQMAQSREDFAKLYDNRV-------
DmLamin_A     208 QETLSRVDLENTIQSLREELSFKDQIHSQEINESRRIKQ-----TEYSEIDG-RLSSEYDAKLKQSLQELRAQYEEQMQINRDEIQSLYEDKI-------
AngLamin_A    211 DETLTRVELENSVQTLREELTFKDQVHQQEITETKTRRQ-----VEISEIDG-MLSEQYEAKLQQTLQDLRDQYDMQLRVNREEISDLYEA--------
CeLamin       199 DETLLRTAANNKIKALEEDLAFALQQHKGELEEVRHKRQ-----VDMTTYAK-QINDEYQSKLQDQIEEMRAQFKNNLHQNKTAFEDAYKNKL-------
MnlLamin      361 RETLKRIDIENRTQTLREEMEFKDSLKEKELTELRRETSST---HIRKMRKK-EAAEEFAVQLSEALAKAREHFDEEKRVLREQLNDVHKKEL-------
AcdLamin      184 QETLSRVDLENKLQSKDEELNFKKEMYNKEILDIRSQLKTVE--TQHIKIAS-DTKSRYEGMLAEKLQELRDLYDTEARQYRDETDSLYSSKY-------
CoLamin       166 KEAQARIAAENALARFKQDAQASAKTGADLNAYHSEEMRRLR--SDLSSLER-KLRAEHDQELAEALSEHRRQTEAESAQYRAEVQALYESRL-------
CrtLamin      227 DAVKNASEAEARRISIEEDLAIERTKAEKASQRFDEEMKQLS--NIDKDVEN-QLRQEFTNQLNSILAERQNQYEAEKNALANELRTLYDEKV-------
Phr_aLamin    158 GHVAALSDATQELEELRKKSADFDLTRDAEMTKTRREWA-----TKHLEAQA-LWKKDAEDRLHTMEMEVRSHFDNVVTSLQNQ---------------
DdLamin       287 ENEYNRFKGKEE-EYNQLIVSKDEEIKKYKFELKEKEKS-----SNAMNKKE-NELNNLIQAHERQIEDMRDSINREWELKAAQMMEEHHART-------

HsVimentin    212 NASLARLDLERKVESLQEEIAFLKKLHEEEIQELQAQIQEQH---VQIDVDVS------KPDLTAALRDVRQQYESVAAKNLQEAEEWYKSKF-------
HsNF-L        201 EAALARAELEKRIDSLMDEISFLKKVHEEEIAELQAQIQYAQ---ISVEMDVT------KPDLSAALKDIRAQYEKLAAKNMQNAEEWFKSRF-------
MdPeripherin  203 DATLSRLELERKIESLMDEIEFLKKLHEEELRDLQVNLESQQ---VQVEVEATV-----KPELTAALRDIRTQYESIAVKNLQEAEEWYKSKY-------
HsKeratin18   191 DTNITRLQLETEIEALKEELLFMKKNHEEEVKGLQAQIASSG---LTVEVDAPKS-----QDLAKIMADIRAQYDELARKNREELDKYWSQQI-------
XtNem         167 EEHSKRLQLHDQVLDLTGRMDQMSDKHRQESEEMRDRIDQSEALVLQLEQKL-RAATGGGSALMEAVQKIQEASEAEVKRLQQETESAYSLSL-------
NvNem         169 TEMAARVDLQTLVNQLREKINFSQQLHEKELAEMRARLDEADRTLMLAEERL-HEHDIIDENLSAMLAKVKLHSDHELRRFKEESEISHINSM-------
SckNem_A      173 KETLAKNEALNALDNLQKKLDFHMQVNAEEAAEMKSRLEDKAKQILMLEGKV-RELGRSDNTLHELLARVRDTAHAELQKYKEESEAQYAKNL-------
CeIF_A        215 QETLNRIDYQNQVQTLLEEIDFLRRVHDNEIKELQTLAS---------RDTTPENREFFKNELSSAIRDIREEYDQVNNVHRNDMESWYRLKV-------
LpIF_A        250 NETINHLDAENRRQTLEEELEFQKDVHAQELKELAALAY---------RDTTAENREFWRNELAQAIRDIQQEYDAKCDQMRGDIEAYYNLKV-------
```

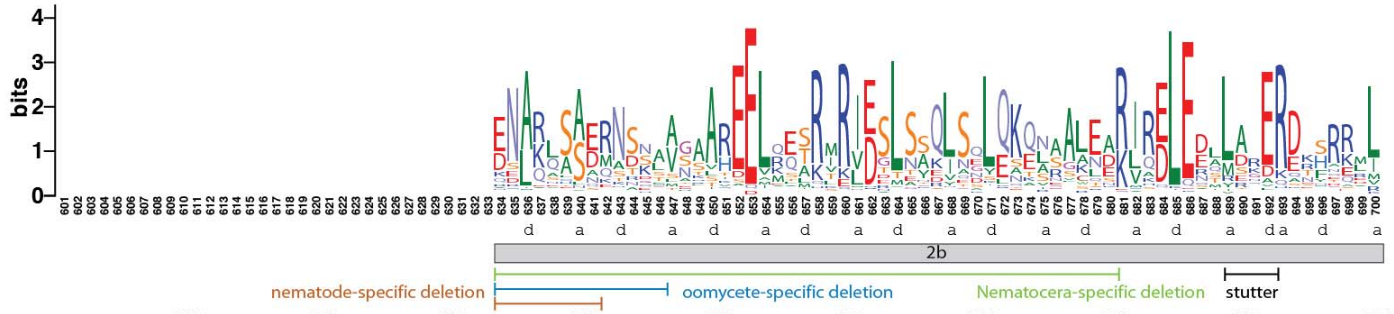


```
                                  610       620       630       640       650       660       670       680       690       700
HsLamin_A       271  ---------------------------------DNARQSAERNSNLVGAAHEELQQSRIRIDSLSAQLSQLQKQLAAKEAKLRDLEDSLARERDTSRRLL
AlsiLamin_A     269  ---------------------------------ENAKQSAERNSNMVGAAHEELQQCRIRIDSLSAQLSQLQKQLAAKEAKLHDLEDALTRERDTSRRIL
HsLamin_B1      272  ---------------------------------ENARLSSEMNTSTVNSAREELMESRMRIESLSSQLSNLQKESRACLERIQELEDLLAKEKDNSRRML
HsLamin_B2      266  ---------------------------------DSAKLSSDQNDKAASAAREELKEARMRLESLSYQLSGLQKQASAAEDRIRELEEAMAGERDKFRKML
AocLamin_E      287  ---------------------------------ESARLCCDQRGKAAGAAEEELAEARLRIRSLGEQLRALQERAAAAEERLREAEGRLSAEREAFRAAL
BrLamin_LIII    280  ---------------------------------ENAKQTAVKNSDFAFSTKEELAGTKLRLESQTQQINNLQKQVVCLEARVLELQQTLEHERQVSQQKL
XlLamin_LIIIb   278  EVLEQQGETKELKNLSECEIQEQVDAMEEQDQLENAQLAAAKNSDYASATREEIMATKLRVDMLTSQLGKYQRQNSALEVKVRELQDMLDRAHEVHRRQM
PtmLamin_C      329  ---------------------------------EKSILTPEKQS-LDISIREDLREATSRVGTLSSQLSAAQHDLALLQGRVRELEETLSHERDQSKRRL
SmLamin         290  ---------------------------------GQLKSSANFSAEDASRQRSELLIARKRADELSHDLSKKIAELELLQRRVADLERQLADERKDFESQL
LhsLamin_C      523  ---------------------------------RDLQTSLSEERGSNASGVQELKESKTRIETLISKVSDLEGFNLNLNQKISDLSQAMDDIKSTHRAQM
DmLamin_A       294  ---------------------------------QRLQEAAARTSNSTHKSIEELRSTRVRIDALNANINELEQANADLNARIRDLERQLDNDRERHGQEI
AngLamin_A      295  --------------------------------------------------------------------------------RLRDLEQQMNHERLRHADEK
CeLamin         285  ----------------------------------------NAARERQEEAVSEAIHLRARVRDLETSSSGNASLIERLRSELDTLKR-------SFQEKL
MnlLamin        449  ---------------------------------DAARAMPRQVVKETTEHTVEIRKYLSEIEELKLTIDAMLAKEKKLQERIKRLEELLKKAEDDAREKD
AcdLamin        273  ---------------------------------DELHKMSSKDLEELTLVREERRALSITVENLRSELNQLKSKNDTLLLRIEDLEKLRASDQESADQAI
CoLamin         255  ---------------------------------EAAREQAVKDASVVAGLQQALSAAQAAQDRARAEVTALSQRSETLSTRVRTLEHELIIERDTVVEVQ
CrtLamin        316  ---------------------------------SAYRAQVEKSSQEKAQLEAECKRFDLETQAAQAEVNKAIADRNSLIAQIQALQQELSQARDGPEREI
Phr_aLamin      235  ----------------------------------------------LDDVRTELDSTKKELDRTANDYEDSLKARQSLTEKVAQLERDYRVERAKSTKDR
DdLamin         372  ---------------------------------IHLQQAVDSFNEEKERIKSQMETLNGQIEDINIKNNEYEDRIKEMNVLLSQKDNSIGELGVEIEESK

HsVimentin      295  ---------------------------------ADLSEAANRNNDALRQAKQESTEYRRQVQSLTCEVDALKGTNESLERQMREMEENFAVEAANYQDTI
HsNF-L          284  ---------------------------------TVLTESAAKNTDAVRAAKDEVSESRRLLKAKTLEIEACRGMNEALEKQLQELEDKQNADISAMQDTI
MdPeripherin    287  ---------------------------------ADLSDAANRNHEALRQAKQEMNESRRQIQSLTCEVDGLRGTNEALLRQLRELEEQFALEAGGYQAGA
HsKeratin18     275  ---------------------------------EESTTVVTTQSAEVGAAETTLTELRRTVQSLEIDLDSMRNLKASLENSLREVEARYALQMEQLNGIL
XtNem           258  ---------------------------------LQLQMQMNSDQRLLSRTQEENQNLQRRLEELSGELALTERKLFSEESNGRTLVGRLESEHRREGQRL
NvNem           260  ---------------------------------NQLKVQLEAESKNLEVATDENIRMKAQLETLQSRIINADAKCASLEAQNASLIQALEMERQQAANTI
SckNem_A        264  ---------------------------------HELQIQMEQDAENIARLADENKKLCEEIDNYRREIHTLQNKVRTLEHNNGDLSESLDQERQKSSVHI
CeIF_A          298  ---------------------------------QEIQTQSARQNMEQGYAKEEVKRLRTQLSDLRGKLADLESRNSLLEKQIQELNYQLEDDQRSYEAAL
LpIF_A          333  ---------------------------------QEFRTGATKQNMEVTRNKEENTKLKSNMTEIRNRLADLEARNAQLERTNQDLLRDLEEKDRQNELES
```

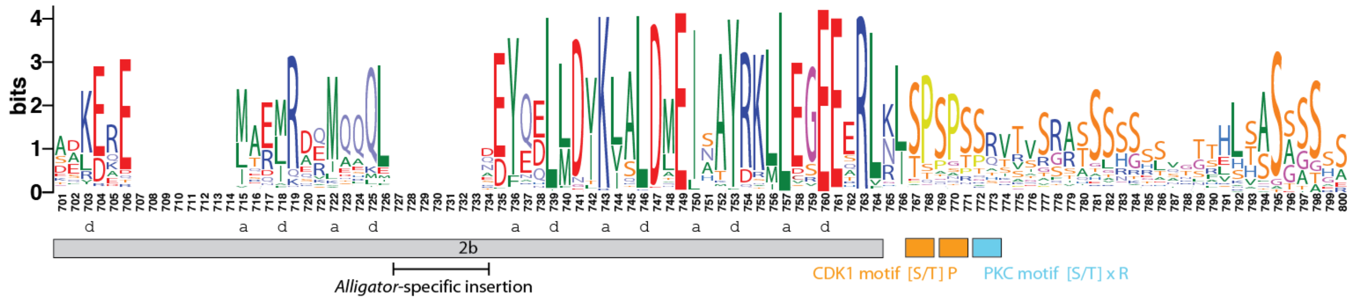


```
                          710       720       730       740       750       760       770       780       790       800
                     ....|....|....|....|....|....|....|....|....|....|....|....|....|....|....|....|....|....|....|....|
HsLamin_A       339  AEKERE--------MAEMRARMQQQL-------DEYQELLDIKLALDMEIHAYRKLLEGEEERLRLSPSPTSQ-RSRGRASSHS----------------
AlsiLamin_A     337  ADKERE--------MADMRARMQQQLEEDQELLDEYQELLGIKLALDMEIHAYRKLLEGEEERLHLSPSPTSQ-KSGSRSHV------------------
HsLamin_B1      340  TDKERE--------MAEIRDQMQQQL-------NDYEQLLDVKLALDMEISAYRKLLEGEEERLKLSPSPSSRVTVSRASS-------------------
HsLamin_B2      334  DAKEQE--------MTEMRDVMQQQL-------AEYQELLDVKLALDMEINAYRKLLEGEEERLKLSPSPSSRVTVSRATSSS-----------------
AocLamin_E      355  EAQERE--------GREMRAQREQQL-------GQYQDLLDVKLALDREIGAYRKLLEGEEQRLKLSPSPARL-TVSRATSSSS----------------
BrLamin_LIII    348  SQQQQE--------MAALRLQMQEQL-------EEYENLLDVKLMLDMEINAYRKMLEGEEKRLNQSLSP-----IQQTLS-------------------
XlLamin_LIIIb   378  TEKDRE--------VTEIRQTLQAQL-------EEYEQLLDVKLALDMEINAYRKMLEGEEQRLKLSPSPSQRSTVSRASTGQ-----------------
PtmLamin_C      396  DESDRE--------LAELREVMDEQL-------IEYEQLLDIKLALDMEIAAYRKLLEGEESRLNLSPEKGVQSSVVQTSTSA-----------------
SmLamin         358  LFQRQE--------VNRLKEELEESF-------REFTDLMNTKIALDQEILMYRKMLEGEESRLNLTPAPRDSPFNIPV---------------------
LhsLamin_C      591  NAKDSE--------IKRLLDELSNQL-------KEYQNLQDIKVALDMEIAVFRSLIEVEEDRLGLGGKGRSRRGSINNNSTVV----------------
DmLamin_A       362  DLLEKE--------LIRLREEMTQQL-------KEYQDLMDIKVSLDLEIAAYDKLLVGEEARLNITPATNTATVQSFSQSLR-----------------
AngLamin_A      316  AKMMEE--------VERLRKEISMQL-------QEYQDLMDIKISLDMEIAAYDKLLSSEEDRLKITPGSQTSIFSQSSMSGSL----------------
CeLamin         339  DDKDAR--------IAELNQEIERMM-------SEFHDLLDVKIQLDAELKTYQALLEGEEERLNLTQ--------------------------------
MnlLamin        517  AQHQAD--------IDKLDKQMKALL-------KDYSDLMDVKVTLDMEIAAYRKLLQAEEVRLNLTPSPKSG-RVRSALSFNDGDAP------------
AcdLamin        341  SSRDKQ--------IRELRKRMDSAL-------RDYEDLMGVKTALDIEISTYRKMLEGEESRLNITPPASPVFG--SSMKPSRR---------------
CoLamin         323  RLREEA--------TRLMQEAIAAKD-------SEVRTLTETNVALDKEIAMYRTLLEVEEKRLGEDFTPTRRGGRDRAASTSVVASGATKRTFSETAEE
CrtLamin        384  EHKKRI--------IRRMKEAYQRKE-------AEFDALMDVKIALSMEIKRYRQMLDEEEDRLGI----------------------------------
Phr_aLamin      290  KAYEET--------LERFRSSKVSKE-------REFNELMDVKIALDAEIMKYRRILDREESRVALATPKTK----------------------------
DdLamin         440  KKMRKQ--------MADLKSKDGQIA-------LLQIEINT-KDNKCNTLQTETNRLKSELYSITNQIDPEIPLDPEINSLKELVKGFE-----------

HsVimentin      363  GRLQDE--------IQNMKEEMARHL-------REYQDLLNVKMALDIEIATYRKLLEGEESRISLPLPNFSSLNLRETNLDSLPLVDTHSKRTLLIKTV
HsNF-L          352  NKLENE--------LRTTKSEMARYL-------KEYQDLLNVKMALDIEIAAYRKLLEGEETRLSFTSVGSITSGYSQSSQVFGRSAYGGLQTSSYLMST
MdPeripherin    355  ARLEEE--------LRQLKEEMARHL-------REYQELLNVKMALDIEIATYRKLLEGEESRISVPIHSFASFSMKTSVPEPELSSDTHSRKMVLIKTI
HsKeratin18     343  LHLESE--------LAQTRAEGQRQA-------QEYEALLNIKVKLEAEIATYRRLLEDGEDFNLGDALD-SSNSMQTIQKTTTRRIVDGKVVSETNDTK
XtNem           326  RELESR--------LEELQDVLLAKM-------KELAAFQESNVSLRNELDALRSLLEEEEQQMSSDQFLSSSSS-------------------------
NvNem           328  KNLEGK--------LRELQEALMAKV-------RELGLAYNAHVPLDLELDAFATLLEAEERRLNLAMQNPTPLAPL-------RSRTWHSITTTTPSKI
SckNem_A        332  QSLEAK--------LKEIQDMLIYKL-------QELGSGK--EPPIRAEIESLKVLVEEEENRLHNIERAHTAVPPINTSGITTSLTGHN----------
CeIF_A          366  NDRDSQ--------IRKMREECQALM-------VELQMLLDTKQTLDAEIAIYRKMLEGEENRAGLKQLVEQVVKT------------------------
LpIF_A          401  CQYKEE--------ITKLRGEMESIL-------KELQDLMDIKLSLELEIAAYRKLLEGEESRVGMKQIVEQVVGARPNEAEVLSTILTRSE--------
```

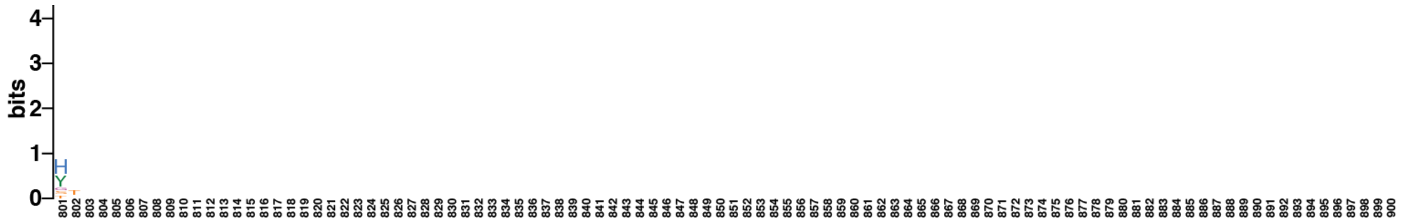


```
                              810       820       830       840       850       860       870       880       890       900
                     ....|....|....|....|....|....|....|....|....|....|....|....|....|....|....|....|....|....|....|....|
HsLamin_A       406  ----------------------------------------------------------------------------------------------------
AlsiLamin_A     409  ----------------------------------------------------------------------------------------------------
HsLamin_B1      405  ----------------------------------------------------------------------------------------------------
HsLamin_B2      401  ----------------------------------------------------------------------------------------------------
AocLamin_E      422  ----------------------------------------------------------------------------------------------------
BrLamin_LIII    408  ----------------------------------------------------------------------------------------------------
XlLamin_LIIIb   445  ----------------------------------------------------------------------------------------------------
PtmLamin_C      463  ----------------------------------------------------------------------------------------------------
SmLamin         421  ----------------------------------------------------------------------------------------------------
LhsLamin_C      659  ----------------------------------------------------------------------------------------------------
DmLamin_A       429  ----------------------------------------------------------------------------------------------------
AngLamin_A      384  ----------------------------------------------------------------------------------------------------
CeLamin         391  ----------------------------------------------------------------------------------------------------
MnlLamin        588  ----------------------------------------------------------------------------------------------------
AcdLamin        408  ----------------------------------------------------------------------------------------------------
CoLamin         408  TN--------------------------------------------------------------------------------------------------
CrtLamin        434  ----------------------------------------------------------------------------------------------------
Phr_aLamin      346  ----------------------------------------------------------------------------------------------------
DdLamin         512  ----------------------------------------------------------------------------------------------------

HsVimentin      448  ETRDGQVINETSQHHDDLE---------------------------------------------------------------------------------
HsNF-L          437  RSFPSYYTSHVQEEQIEVEETIEAAKAEEAKDEPPSEGEAEEEEKDKEEAEEEEAAEEEEAAKEESEEAKEEEEGGEGEEGEETKEAEEEE-KKVEGAGE
MdPeripherin    440  ETRDGEVVTESHKEQRSEIDKSSTHSY-------------------------------------------------------------------------
HsKeratin18     427  VLRH------------------------------------------------------------------------------------------------
XtNem           385  ---------------------------------------------------------------------------------SGPALTHSFLSSDLTIGLD
NvNem           406  TEIKRPASTLGIVPVS------------------------------------------------------------------------------------
SckNem_A        404  ----------------------------------------------------------------------------------------------------
CeIF_A          426  ----------------------------------------------------------------------------------------------------
LpIF_A          477  ----------------------------------------------------------------------------------------------------
```

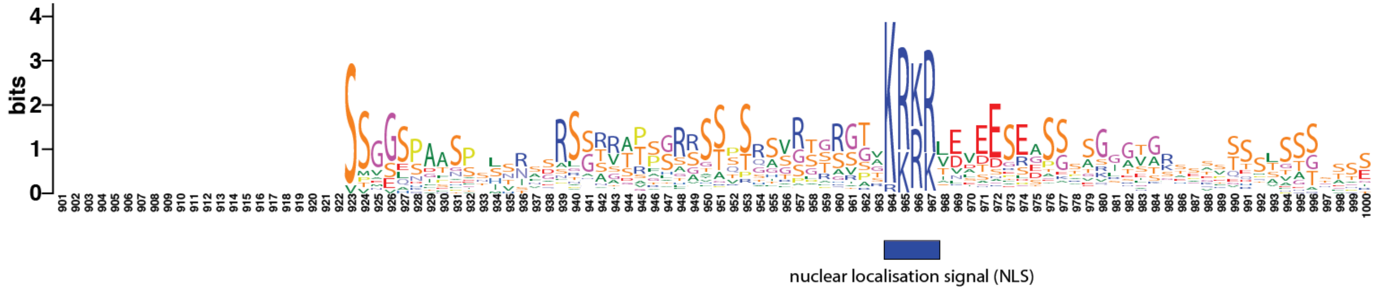


```
                                  910       920       930       940       950       960       970       980       990      1000
                           ....|....|....|....|....|....|....|....|....|....|....|....|....|....|....|....|....|....|....|....|
HsLamin_A          406     --------------------------------------------------SQTQ--GGGSVT-KKRKLESTESR--------------------------
AlsiLamin_A        409     ---------------------------------------------------VRSSMQSSS---KKRKLEDGEGR--------------------------
HsLamin_B1         405     ----------------------------------------------------SRSVRTTRG--KKRKRVDVEESEASS----------------------
HsLamin_B2         401     ------------------------------------------------SGSLSATGRLGRS--KKRKRLEVEEPLGSGPSVLGT----------------
AocLamin_E         422     ------------------------------------------------------SSSSWRG--KKRKRREELPSETSPA---------------------
BrLamin_LIII       408     ------------------------------------------------------PIIQQSR--KKRKHDATCSGFSP-----------------------
XlLamin_LIIIb      445     ------------------------------------------------------TSRLLRG--KKRKLEETDRSVTKR----------------------
PtmLamin_C         463     ----------------------------------------------------SHSIRTMPG--KKRKRVQLEGFVDSMAQSQ------------------
SmLamin            421     ---------------------------------------------------------------KRRRVDGDFEGDESNASLTGA----------------
LhsLamin_C         659     ----------------------------------------------------------------------------------------------------
DmLamin_A          429     ---------------------------------------NSTRATPSRR-TPSAAV-------KRKRAVVDESED-------------------------
AngLamin_A         384     -----------------------------------------SRSGVVRR-TPSRAAA------KRKRTVLEESDE-------------------------
CeLamin            391     -------------------------------------EAPQNTSV-HHVSFSSGG-ASAQRGVKRRRVVDVNGED-------------------------
MnlLamin           588     -------------------------------------------------------------SRKRQRVMEES----------------------------
AcdLamin           408     -------------------------------------------------------------SVKRVRTEEEST---------------------------
CoLamin            409     ---------------------------------------------------------TTPVRSKRRREEDPSPSRSASSSSATFFAGAPRSVVNAAT---
CrtLamin           434     ----------------------------------------------------------------------------------------------------
Phr_aLamin         346     --------------------------------------------------------------GRKRKSENTNGTSSKRTKRALAVAESVTD-LSSS----
DdLamin            512     ---------------------------------------------------------KTVDDRKRKRSKLQHEFNAAANQDQNGMTIEEQSSTSTTTTTS

HsVimentin         466     ----------------------------------------------------------------------------------------------------
HsNF-L             536     EQAAKKKD--------------------------------------------------------------------------------------------
MdPeripherin       466     ----------------------------------------------------------------------------------------------------
HsKeratin18        430     ----------------------------------------------------------------------------------------------------
XtNem              405     CTASPLIGSLDVLSPPTTAKCPKSPSPAESEEPNQNLDEGPKPGGHPRPASAPPLSPNVPPGTDPAAPERRGLTVNRKK---------------------
NvNem              421     ------------------------------------------------------------AVTTVRKPVTRPKTTSGTLSVSKSIPPRPSTEPITTSSPY
SckNem_A           404     ------------------KLPLSYTPGSRLVKCGVSYSGQTRSSPPAYAGKFANTTGMSTSVTSRGYTRPTRPASVPAP-VTGQGKDYFDSMFGDVKRDT
CeIF_A             426     -------------------------------------------------HAITQETDTETM---------------------------------------
LpIF_A             477     ----------------------------------------------------------------------------------------------------
```

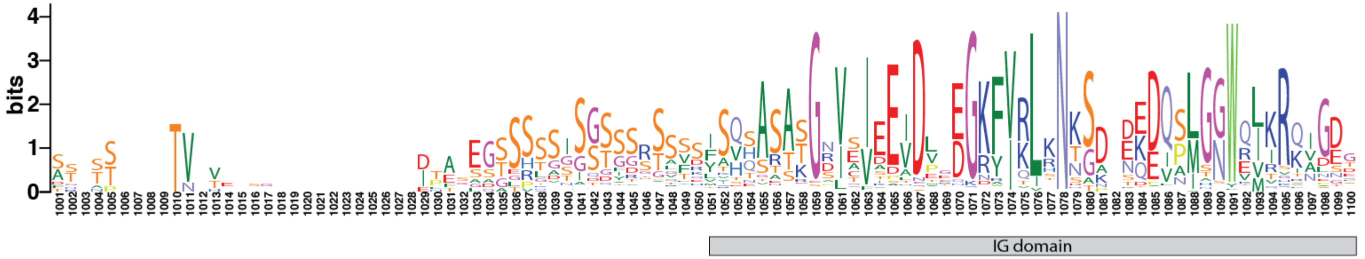


```
                         1010      1020      1030      1040      1050      1060      1070      1080      1090      1100
                    ....|....|....|....|....|....|....|....|....|....|....|....|....|....|....|....|....|....|....|....|
HsLamin_A      427  ------------------------------------------------SSFSQHARTSGRVAVEEVDE-EGKFVRLRNKS--NEDQSMGNWQIKRQNGDD
AlsiLamin_A    429  ------------------------------------------------TTFSHHVRTSGRVAVEEVDL-DGKFVRLKNKS--SEDQALGNWQIKRVNGED
HsLamin_B1     428  -----------------------------------------------SVSISHSASATGNVCIEEIDV-DGKFIRLKNTS--EQDQPMGGWEMIRKIGDT
HsLamin_B2     434  -----------------------------------------GTGGSGGFHLAQQASASGSVSIEEIDL-EGKFVQLKNNS--DKDQSLGNWRIKRQVLEG
AocLamin_E     444  -----------------------------------------------TSASQTDSAPGSNIHIEEVDP-QGTFVRLRNRS--HQDVSLGNWRLKKVGGER
BrLamin_LIII   429  -----------------------------------------------NYRLSQHSSCRGPVSIEEVDL-DGRYIKLKNAS--DRDQVVSGWQLRKSEAEA
XlLamin_LIIIb  467  -----------------------------------------------AYKVVQQASSTGPVSVEDIDP-EGNYVRLLNNT--EEDFSLHGWVVKRKHMSL
PtmLamin_C     490  ------------------------------------------------RSVSQHASASGSIGIKEVDM-EGKSVTMVNNS--EKDQPLGGWVLKRQIEDQ
SmLamin        442  ----------------------------------------SYSSSRTRFAYRVSSTARGPVEFFKEQDTHGKWIKIHNSS--NDEMNLGSWELVHEADGH
LhsLamin_C     659  ----------------------------------------------------------------------------------------------------
DmLamin_A      457  ---------------------------------------------HSVADYYVSASAKGNVEIKEIDP-EGKFVRLFNKG--SEEVAIGGWQLQRLINEK
AngLamin_A     411  ---------------------------------------------RSVSDYSVTSSAKGDVEIVEVDP-EGKYVKLHNKS--GKEVQIGGWTVLRKVGSN
CeLamin        427  -----------------------------------------QDIDYLNRRSKLNKETVGPVGIDEVDE-EGKWVRVANNS--EEEQSIGGYKLVVKAGNK
MnlLamin       599  ----------------------------------------------------FTTETNSHVSIDEVSI-EHDYVGITNMT--EKDQTMKGWTIAKVSGEE
AcdLamin       420  ---------------------------------------------------GTKYSHQGYIQIKEANA-DGKYIKLFNSG--SQDVSLGGWLLERSVDGD
CoLamin        449  --------------RALFTS------------SASSSSDASSSSTGTTLWTSEPVTEAGLVVFEPVNLVEHNKLVLRSTNP-EEEVSLHGWKVRTTSETT
CrtLamin       434  ----------------------------------------------------------------------------------------------------
Phr_aLamin     379  -----------------------------------------------------------SVQIASLDL-EKDRIVLKNSS--SEPVPLGGWVVRGQMDQ-
DdLamin        556  ATGSS---------------------------SSTSHLDNIDSSKLPTGPEQSELFNPDTVSFSLVDS-NQEFIKLSVHGDMDNGLSISKWRLIVVKPDG

HsVimentin     466  ----------------------------------------------------------------------------------------------------
HsNF-L         543  ----------------------------------------------------------------------------------------------------
MdPeripherin   466  ----------------------------------------------------------------------------------------------------
HsKeratin18    430  ----------------------------------------------------------------------------------------------------
XtNem          483  ------------------------------------------PSPNNDCTPAIS-SALRDLAISEVDP-GGKFVKIINKSL-DKEEDIGGHILQQNIGGH
NvNem          462  RHSWSYVPNFVDYHS-------------------------------------PTSSHTGNVRILEVNP-DGNYVRLFNTSA-HRDEEIGGYMIQQNVAGR
SckNem_A       486  LYYPRLHPKSSPPSFT----------------------------PATHDYNTSTSSFTGTIKILEINQ-DGRFVRLFNSSP-NQDMEFGGFMIQQNVGGH
CeIF_A         438  ----------------------------------------RVVKGETASRQSFQRSAKGNVSIHEASP-DGKFIVLQNTHR-AKDEAIGEWKLKRRIDGK
LpIF_A         477  --------------------------GGYEATG-DSQISMKMMRGELAAKTTYQRTSKGSVSIKEADS-QGCFIALET----KKEENLTGWKIVRKVDDN
```

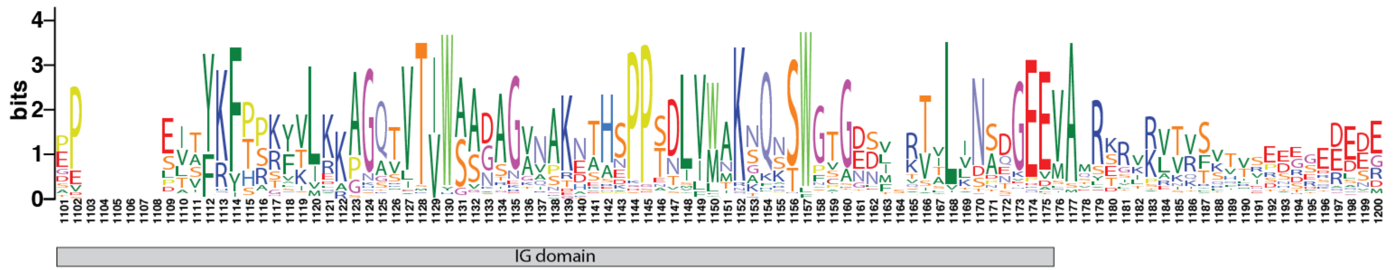


```
                               1110      1120      1130      1140      1150      1160      1170      1180      1190      1200
                          ....|....|....|....|....|....|....|....|....|....|....|....|....|....|....|....|....|....|....|....|
HsLamin_A         477    P-------LLTYRFPPKFTLK-AGQVVTIWAAGAGA----THSPPTDLVW-KAQNTWGCGNSL-RTALINSTGEEVAMRK---LVRSVTVVED---DEDE
AlsiLamin_A       479    P-------VVSYRFPPKFTLK-AGQVVTIWGSGAGA----THSPPTDLVW-KAQSSWGTGDSL-RTALINSNGEEVAMRK---LVRTVIINDE---DEDG
HsLamin_B1        478    --------SVSYKYTSRYVLK-AGQTVTIWAANAGV----TASPPTDLIW-KNQNSWGTGEDV-KVILKNSQGEEVAQRS----TVFKTTIPE---EEEE
HsLamin_B2        491    E-------EIAYKFTPKYILR-AGQMVTVWAAGAGV----AHSPPSTLVW-KGQSSWGTGESF-RTVLVNADGEEVAMRT---VKKSSVMREN----ENG
AocLamin_E        495    GEAAT---ADAYKFGPRFVLR-AGQSVTVWSSDAGG----PPHPPGALVWRGRSRPWGPGDSA-RLLLTNTHGQEVATRS---------LSPEEEEEEEG
BrLamin_LIII      480    P-------DIVFCFPSPCVMN-AGHMLTVWAAAAVD----QCGSASDLLL-STHQSWGSFADV-RVSLLNIQNEEMAEYY-----VVQD--------KTE
XlLamin_LIIIb     518    P-------DISFKLPCRFILK-SSQHVTIWATGAGA----VHTPPTDLVW-KSQKTWGTGDNI-KITLHDSSGEECAERT-----LYRVIGE------EG
PtmLamin_C        540    A-------EIAYKFSSRYVLK-AGQTVTIWAANAGV----SHNPPSDLLW-KTQSSWGTGDDI-RTSLLNSDGEEVAVRN---VVRSSKVADK-------
SmLamin           500    --------ETRFKFSRAFSLK-PGAICTIWSQDAEGN---SHNPPADLTM-KNKS-FHPGTEV-TILLLDTDGEEQAKCTVKREKIRPG-VNFDRKSGVR
LhsLamin_C        659    ----------------------------------------------------------------------------------------------------
DmLamin_A         510    GP------STTYKFHRSVRIE-PNGVITVWSADTKA----SHEPPSSLVM-KSQK-WVSADNT-RTILLNSEGEAVANLDRIKRIVSQHTSSS---RLSR
AngLamin_A        463    --------ETVFKFHRSLKMD-GGAFVTVWSSDLGK----DHEPPTTIVM-KGQK-WFAGDNM-TTHLLNAEGEEVAASERVK----RMVSTA----ASR
CeLamin           483    --------EASFQFSSRMKLA-PHASATVWSADAGA----VHHPPEVYVM-KKQQ-WPIGDNP-SARLEDSEGDTVSSITV-------------------
MnlLamin          644    --------TLEYKFPAKAVIG-PGQTLKVYSGGAGA----THNPPLSLVF-KKLDAWSNTMDL-EIVLRNSDGVVVSSYKAKA------------------
AcdLamin          467    P-------AIVYKFTPKYVLK-SGSYVTVWASQGGG----QHKPPTDLVF-RQKASWGVGRET-RTLLKDADGQEAASAVSEEVSIVRTDEIDTR-----
CoLamin           523    G-------SATYEIPAGIALSAEHPTVTIWTGVSATRG--KRKSRSELLW-KQDINYASNGAC-IQLLDNEDNEIAR-----------------------
CrtLamin          434    ----------------------------------------------------------------------------------------------------
Phr_aLamin        416    ----------TFRFPATYVMR-PQSTLTVHSSKRNKNAKNERKKGEDSFLANKFS-LNPDGDF-VILVTSDDVPVSMESEGLPE----------------
DdLamin           628    S-------KSGFSFPDGIQPFKGIKSVTVWTGRPRP----QGTPTENEFY------WARTELW-TSPVEGTIVKLVSPS---------------------

                                                                                                  Nematocilin tail domain
HsVimentin        466    ----------------------------------------------------------------------------------------------------
HsNF-L            543    ----------------------------------------------------------------------------------------------------
MdPeripherin      466    ----------------------------------------------------------------------------------------------------
HsKeratin18       430    ----------------------------------------------------------------------------------------------------
XtNem             539    P-------VALYRFPPKTRML-AHSEVTVWAASANI----PHKPPSDFLW-KDLDEFVTGPQC-TTILCKANGQAVAWYTPARGTCSPPVGRKGDTTATP
NvNem             523    P-------VTVFRFPPRTRLK-ALSHTTVWSAGSLS----RHNPPSDFLW-KEQHKWGTGPEC-TTILCKPNGQAMAWTTAAFPFGVPNQIPTRDTGPTS
SckNem_A          556    P-------VAVYRFPPRTRFR-AGSTITVWAASGQG----KHSPPTDFLW-KEQHKWGVGPEC-TTILCKPTGQAIAWTTAAHRFTRNAISFDAVSDNSS
CeIF_A            497    R-------ENVYTLPRDFVLR-AGKTLKIFARNQG-----VASPPDQLVY-DAEDSFGSGNNVQTILFNKEGE-----ERATHIQRQSTA----------
LpIF_A            546    K--------VYTYEIPNLVLK-TGTVVKIWSKNHQA-----QARGDDLVSR-ENDTWGTGSNVVTILQNEKGE-----DKANYTQNTVYQ----------
```

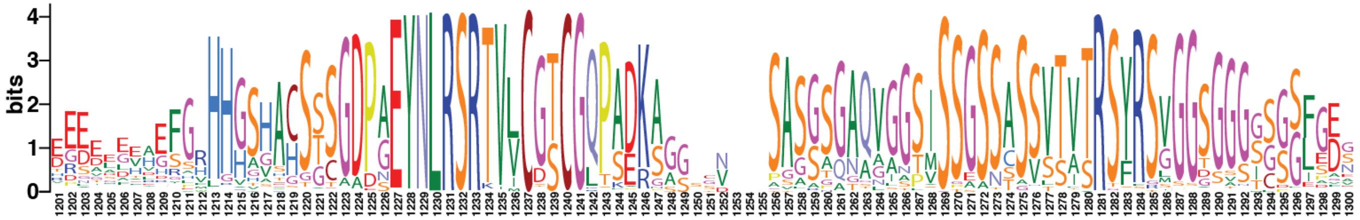

vertebrate lamin A-specific insertion

```
                     1210      1220      1230      1240      1250      1260      1270      1280      1290      1300
                 ....|....|....|....|....|....|....|....|....|....|....|....|....|....|....|....|....|....|....|....|
HsLamin_A    557 DGDDLLHH----HHGSH-CSSSGDPAEYNLRSRTVLCGTCGQPADKA--------SASGSGAQVGGPISSGSSASSVTVTRSYRSVGGSGGG---SFGDN
AlsiLamin_A  559 DEEGMHPHR---HHHVH-CSGSGDPGEYNLRSRTVVCGTCGQPAEKS-----------GTGTQNAGTVSSGSSAASVTITRSYRSLGGTGGSTS-GLGES
HsLamin_B1   557 EEEAAGVV--------------------------------------------------------------------------------------------
HsLamin_B2   570 EE-EEEEAEF------------------------------------------------------------------------------------------
AocLamin_E   577 EEEEA----------------------------------------------------------------------------------------------Q
BrLamin_LIII 553 EDAESD-------------------------------------------------------------------------------------------EPV
XlLamin_LIIIb 593 ETDEDAEE--------------------------------------------------------------------------------------------
PtmLamin_C   616 PDEEVMQY--------------------------------------------------------------------------------------------
SmLamin      585 A---------------------------------------------------------------------------------------------------
LhsLamin_C   659 ----------------------------------------------------------------------------------------------------
DmLamin_A    593 RRSVTAVD--------------------------------------------------------------------------------------------
AngLamin_A   540 HREYLGSY--------------------------------------------------------------------------------------------
CeLamin      548 ----------------------------------------------------------------------------------------------------
MnlLamin     712 ----------------------------------------------------------------------------------------------------
AcdLamin     547 ----------------------------------------------------------------------------------------------------
CoLamin      588 ----------------------------------------------------------------------------------------------------
CrtLamin     434 ----------------------------------------------------------------------------------------------------
Phr_aLamin   487 ----------------------------------------------------------------------------------------------------
DdLamin      688 --------------------------------------------------------------------------------------------------EE
```

Nematocilin tail domain

```
HsVimentin   466 ----------------------------------------------------------------------------------------------------
HsNF-L       543 ----------------------------------------------------------------------------------------------------
MdPeripherin 466 ----------------------------------------------------------------------------------------------------
HsKeratin18  430 ----------------------------------------------------------------------------------------------------
XtNem        625 NNSSLLWTLP-----------------QPNGNRESEPESPKQVPERTNDQQKWEEGPILLRREKL-PPAVLTPTCSPWTQSVASPTHPDFSPSQSWGTES
NvNem        609 PRDSGEQLNNENTGPM-------------------------------------------SPRYKTPEPIPREIEGKPFSRPPTDPLHPHSVQSRVKAGGH
SckNem_A     642 DADKGESNPSP---------------------------------EFTVEVD-GKP-VTALKVEKEEQPLLSPAKHPHGLF-TGDDPHPSMGAPRVKAGGN
CeIF_A       567 ----------------------------------------------------------------------------------------------------
LpIF_A       615 ----------------------------------------------------------------------------------------------------
```

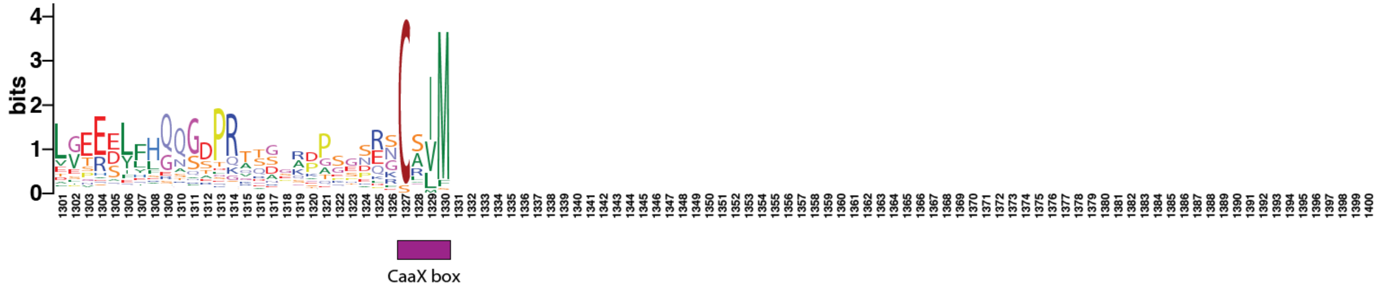


```
                              1310      1320      1330      1340      1350      1360      1370      1380      1390      1400
                         ....|....|....|....|....|....|....|....|....|....|....|....|....|....|....|....|....|....|....|....|
HsLamin_A          641   LVTRSYLLGNSSPRTQS------PQNCSIM----------------------------------------------------------------------
AlsiLamin_A        643   LVTRSYILGNASPRRQ------------------------------------------------------------------------------------
HsLamin_B1         564   -VEEELFHQQGTPRAS-------NRSCAIM----------------------------------------------------------------------
HsLamin_B2         578   -GEEDLFHQQGDPRTT-------SRGCYVM----------------------------------------------------------------------
AocLamin_E         583   DFGQEDLFHQQGDPRTT------SKGCAVM----------------------------------------------------------------------
BrLamin_LIII       562   RSRDAHMRRQGQTLDH-------DSACLLM----------------------------------------------------------------------
XlLamin_LIIIb      600   -EEFERQFRSQSHQSV-------DPSCSIM----------------------------------------------------------------------
PtmLamin_C         624   TREEPLQYQVESTS---------SKRCLIM----------------------------------------------------------------------
SmLamin            585   ----------------------HDEKCHLM----------------------------------------------------------------------
LhsLamin_C         659   ----------------------------------------------------------------------------------------------------
DmLamin_A          600   -GNEQLYHQQGDPQQS-------NEKCAIM----------------------------------------------------------------------
AngLamin_A         547   --GEELHHQQGEPRG--------DEKCRIM----------------------------------------------------------------------
CeLamin            548   -----EFSESSDPSDP-------ADRCSIM----------------------------------------------------------------------
MnlLamin           712   ----ETSTEYLEEGD--------QRACSVM----------------------------------------------------------------------
AcdLamin           547   ------------PVMYAKSDGETGKGCLIQ----------------------------------------------------------------------
CoLamin            589   LEVREQKEDELFERQSDPR-----VGCSIM----------------------------------------------------------------------
CrtLamin           434   ----------------------------------------------------------------------------------------------------
Phr_aLamin         488   EEVRAIESELRADFMD--DDAPPSGNCGMM----------------------------------------------------------------------
DdLamin            691   TTTVTLPADGIYQKPSSAGK----SNCLIM----------------------------------------------------------------------

                         [Nematocilin tail domain]

HsVimentin         466   ----------------------------------------------------------------------------------------------------
HsNF-L             543   ----------------------------------------------------------------------------------------------------
MdPeripherin       466   ----------------------------------------------------------------------------------------------------
HsKeratin18        430   ----------------------------------------------------------------------------------------------------
XtNem              707   DGFSKCHLPGPQRSYVAPSPGSRRRKEPITEPKFRSSRGPTRSAR------------------SSAKGVLYLGYSSPAGSTLQKYMGNASYDIRLASHSS
NvNem              666   DGMTVNPQSR--SQTTRPDPA---------------GATRTGGAPLRRYAGTSLPNDKDSSSKQTHGGTIRVVPATNFSSPSKQHQDRLANLSANHKVDF
SckNem_A           706   DNSSYCRQTR--SQNSNPVPDRMPHPGDLYAGAGA-GGTRLGSAPLRSSSAMKANN------------GSKSTQLSPFMSPHQQKYSTVNKISSQHRVTF
CeIF_A             567   ----------------------------------------------------------------------------------------------------
LpIF_A             615   ----------------------------------------------------------------------------------------------------
```

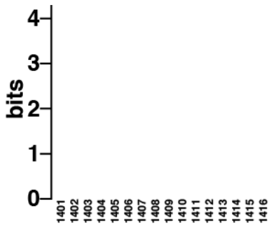

Supplementary Figure S1: The sequence logos illustrate the sequence conservation within the multiple sequence alignment of 629 lamins. For better orientation, the sequences of several representative lamins and cytoplasmic IF proteins are shown. The heptat positions "a" and "d" refer to HsLamin_A and have been determined with Marcoil. The C-terminal exon is missing in the AlsiLamin_A sequence due to a sequencing gap. The CrtLamin sequence was derived from a TSA assembly and is not complete, missing almost the complete lamin tail sequence.

```
                          1410
                    ....|....|....|.
HsLamin_A      664  ----------------
AlsiLamin_A    658  ----------------
HsLamin_B1     586  ----------------
HsLamin_B2     600  ----------------
AocLamin_E     606  ----------------
BrLamin_LIII   584  ----------------
XlLamin_LIIIb  622  ----------------
PtmLamin_C     644  ----------------
SmLamin        593  ----------------
LhsLamin_C     659  ----------------
DmLamin_A      622  ----------------
AngLamin_A     567  ----------------
CeLamin        566  ----------------
MnlLamin       730  ----------------
AcdLamin       565  ----------------
CoLamin        613  ----------------
CrtLamin       434  ----------------
Phr_aLamin     515  ----------------
DdLamin        716  ----------------

HsVimentin     466  ----------------
HsNF-L         543  ----------------
MdPeripherin   466  ----------------
HsKeratin18    430  ----------------
XtNem          789  LSPGDLSGVSRMRHTL
NvNem          749  SPPAPRPYVATR----
SckNem_A       791  QPPMPRPPVVSSW---
CeIF_A         567  ----------------
LpIF_A         615  ----------------
```

Hs	*Homo sapiens*
Alsi	*Alligator sinensis*
Aoc	*Anolic carolinensis*
Br	*Brachydanio rerio*
Xl	*Xenopus laevis*
Ptm	*Petromyzon marinus*
Sm	*Schistosoma mansoni*
Lhs	*Lepeophtheirus salmonis*
Dm	*Drosophila melanogaster*
Ang	*Anopheles gambiae*
Ce	*Caenorhabditis elegans*
Mnl	*Mnemiopsis leidyi*
Acd	*Acropora digitifera*
Co	*Capsaspora owczarzaki*
Crt	*Corallomyxa tenera*
Phr_a	*Phytophthora ramorum strain EU2_996_3*
Dd	*Dictyostelium discoideum*
Md	*Monodelphis domestica*
Xt	*Xenopus tropicalis*
Nv	*Nematostella vectensis*
Sck	*Saccoglossus kowalevskii*
Lp	*Loligo pealeii*

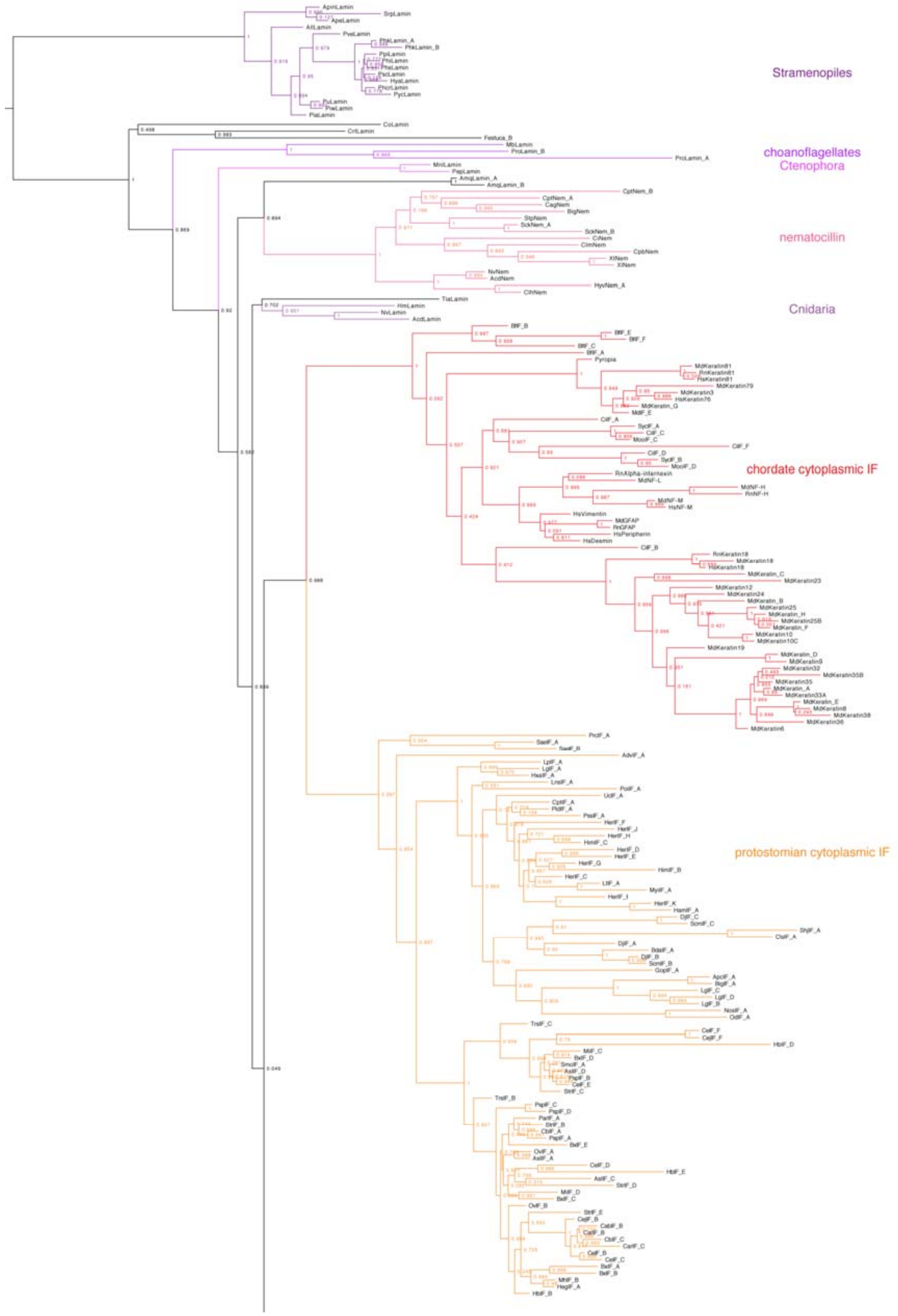
Stramenopiles
choanoflagellates
Ctenophora
nematocillin
Cnidaria
chordate cytoplasmic IF
protostomian cytoplasmic IF

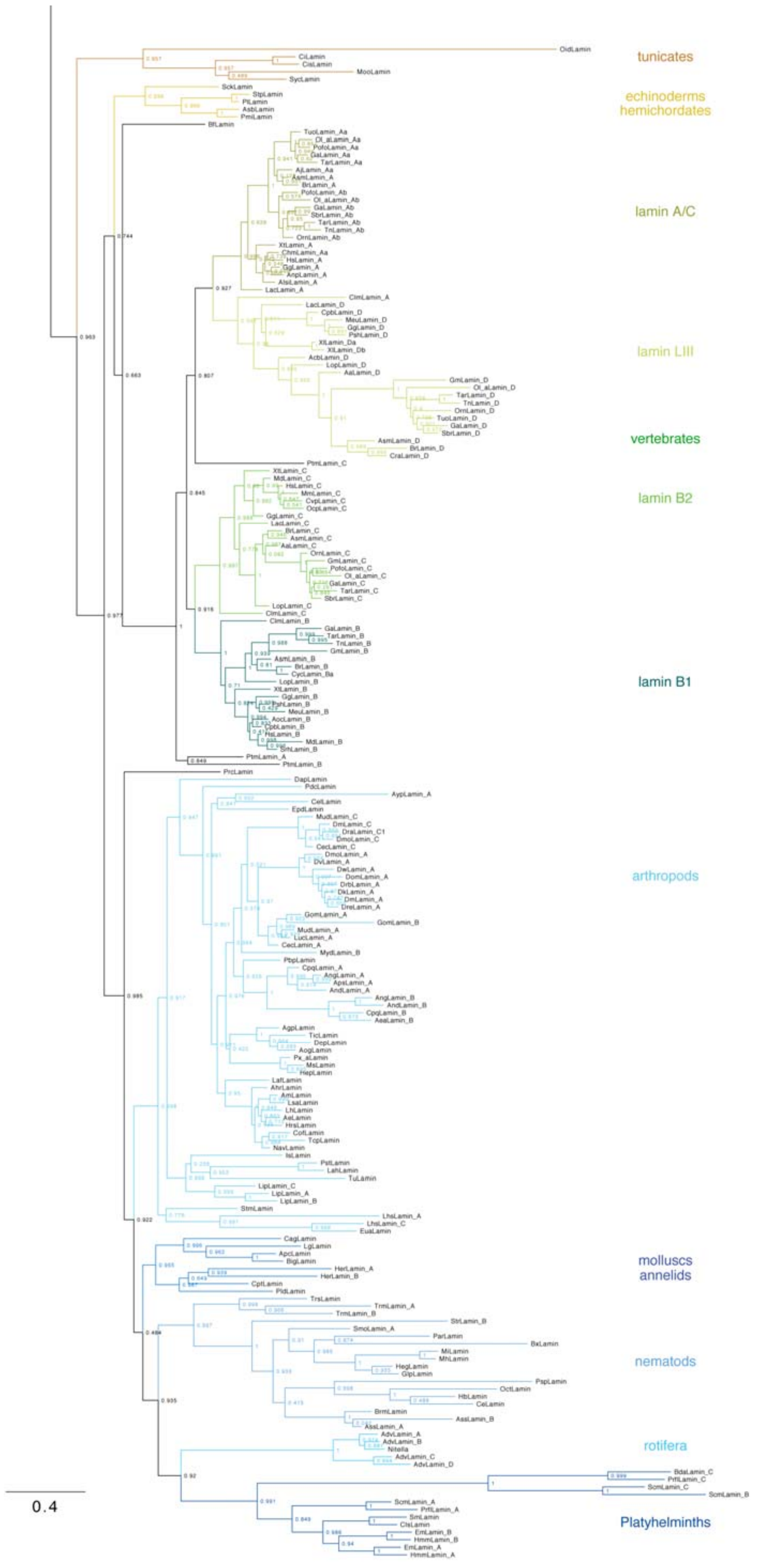
tunicates
echinoderms
hemichordates
lamin A/C
lamin LIII
vertebrates
lamin B2
lamin B1
arthropods
molluscs
annelids
nematods
rotifera
Platyhelminths
0.4

Supplementary Figure S2. Phylogeny of the intermediate filament proteins. Maximum-likelihood topology generated under the JTT + Γ model in FastTree showing branch lengths for 232 lamins, 16 nematocilins, and 145 cytoplasmic IF proteins. CD-Hit (90% idenity) was used and the amoebozoan lamins as well as highly variable regions were removed manually to obtain a representative dataset for subtype classification and visualization. This tree is identical to the tree in Fig. 1 of the main manuscript but contains the support values for all branchings given as likelihood bootstrap. The scale bar corresponds to estimated amino acid substitutions per site.

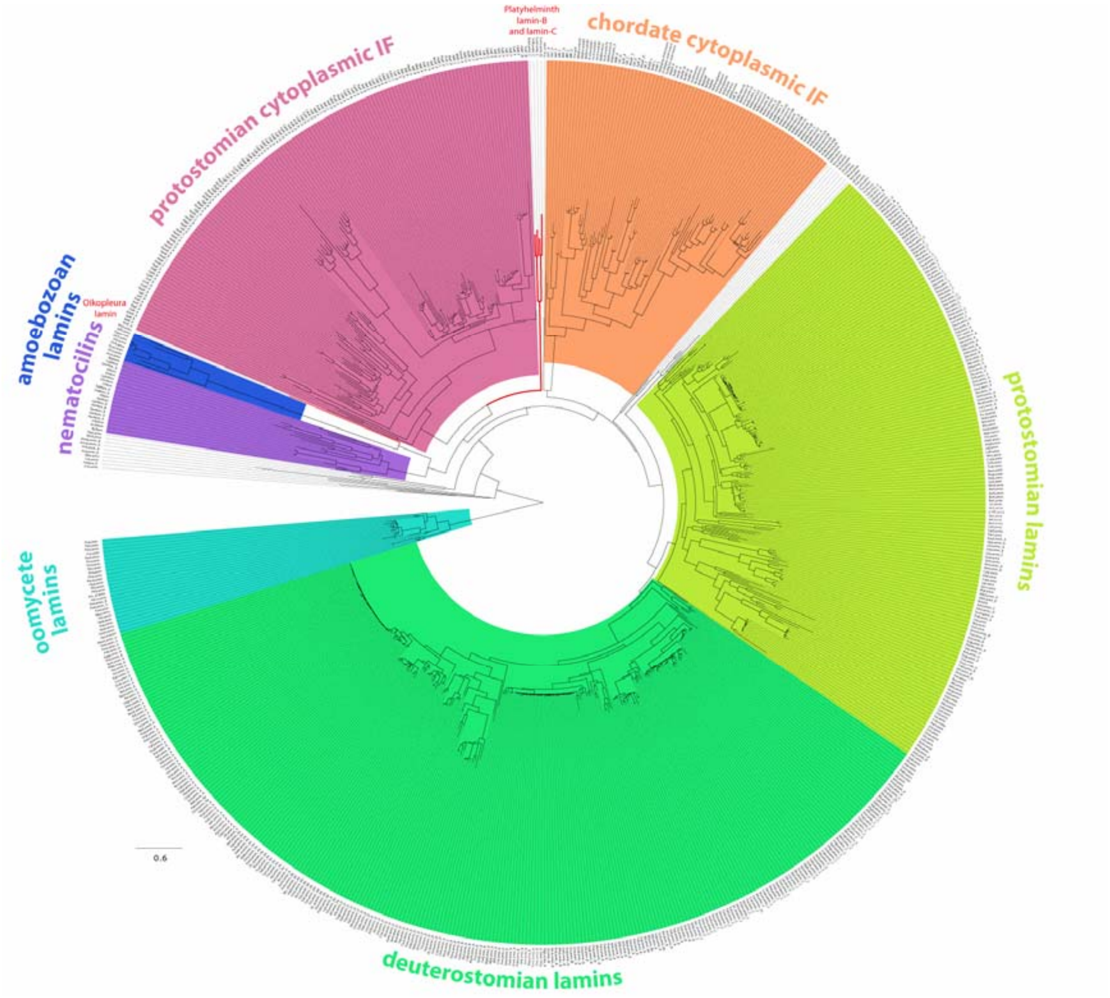

Supplementary Figure S3. Phylogeny of the intermediate filament proteins. Maximum-likelihood topology generated under the JTT + Γ model in FastTree showing branch lengths for 492 lamins, 20 nematocilins, and 217 cytoplasmic IF proteins. The scale bar corresponds to estimated amino acid substitutions per site. Major IF groups are colored and the *Oikopleura dioica* (*Oid,* Tunicate) and a group of Platyhelminth lamins highlighted in red. Most probably because of branch-attraction effects the amoebozoan lamins and the *Oid*Lamin group to the nematocilins and the Platyhelminth lamins to the protostomian cytoplasmic IF proteins.

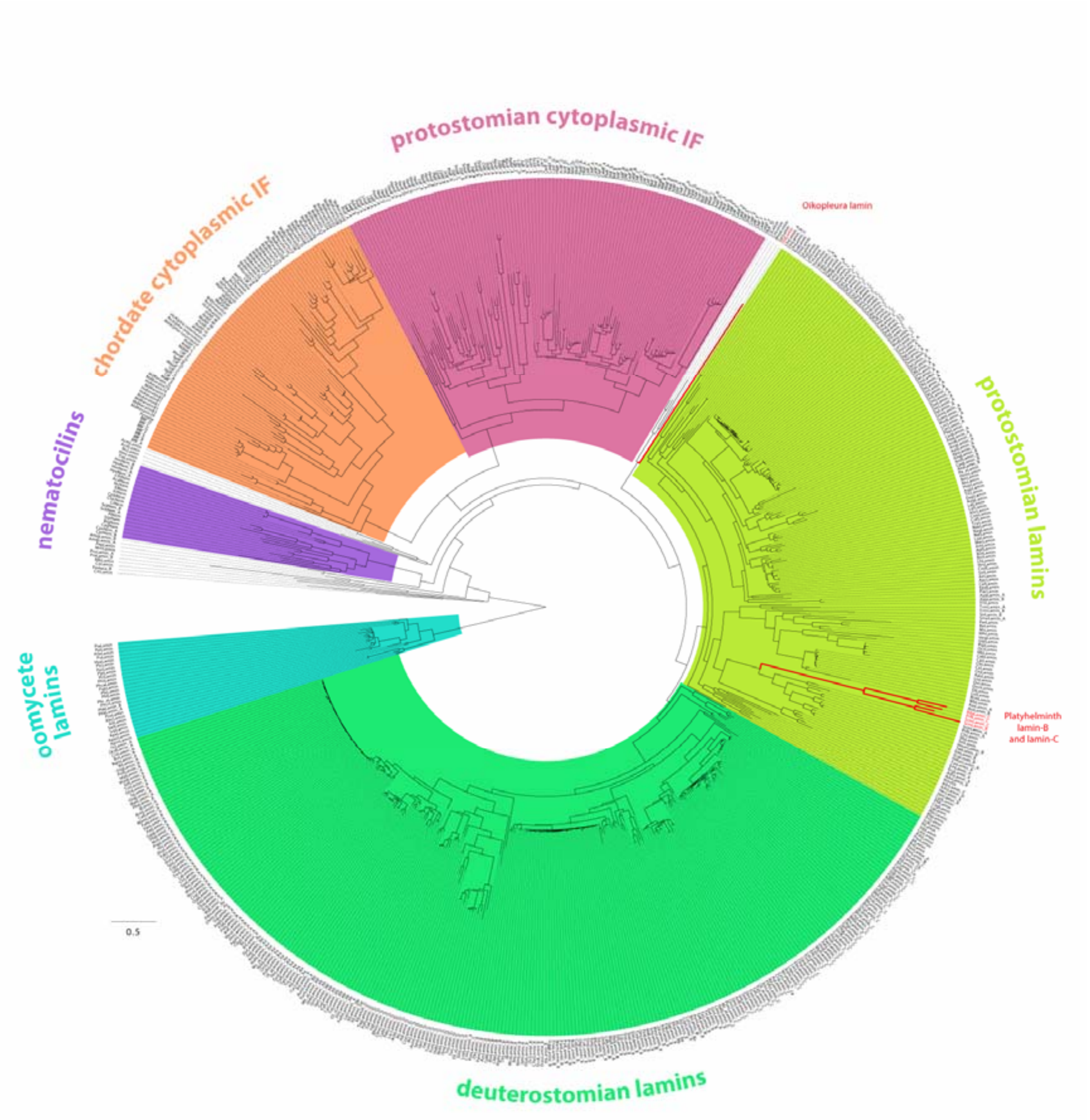

Supplementary Figure S4. Phylogeny of the intermediate filament proteins without amoebozoan lamins. Maximum-likelihood topology generated under the JTT + Γ model in FastTree showing branch lengths for 484 lamins, 20 nematocilins, and 198 cytoplasmic IF proteins. Compared to the dataset shown in fig. S3 the amoebozoan lamins were removed. The scale bar corresponds to estimated amino acid substitutions per site. Major IF groups are colored and the *Oikopleura dioica* (*Oid*, Tunicate) and a group of Platyhelminth lamins highlighted in red. The *Oid*Lamin groups to the other tunicate lamins and the Platyhelminth lamins of type-B and type-C to the type-A Platyhelminth lamins.

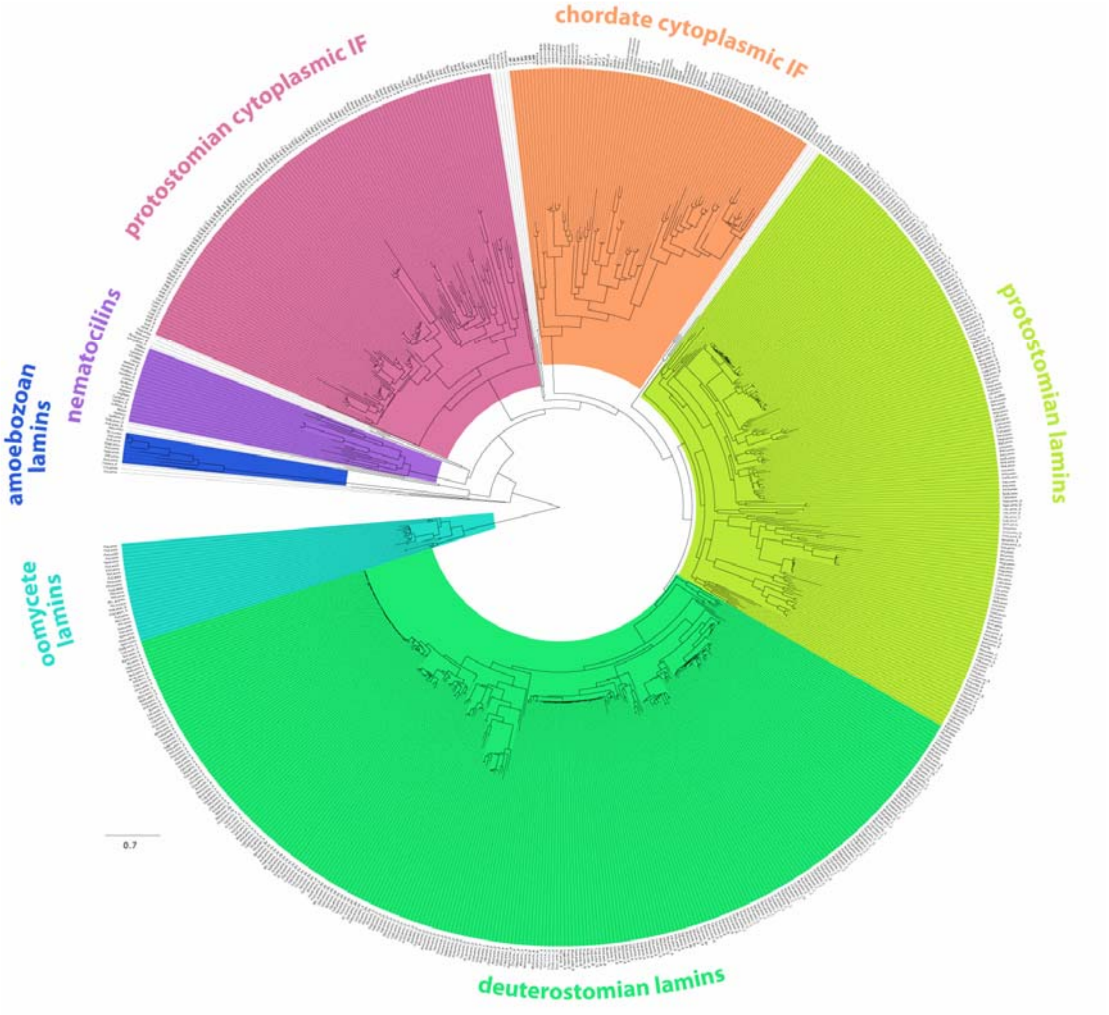

Supplementary Figure S5. Phylogeny of the intermediate filament proteins without *Oid*Lamin and subtype-B and -C Platyhelminth lamins. Maximum-likelihood topology generated under the JTT + Γ model in FastTree showing branch lengths for 487 lamins, 20 nematocilins, and 198 cytoplasmic IF proteins. Compared to the dataset shown in fig. S3 the *Oid*Lamin and the subtype-B and -C Platyhelminth lamins were removed. The scale bar corresponds to estimated amino acid substitutions per site. Major IF groups are colored. The amoebozoan lamins group outside the metazoans.

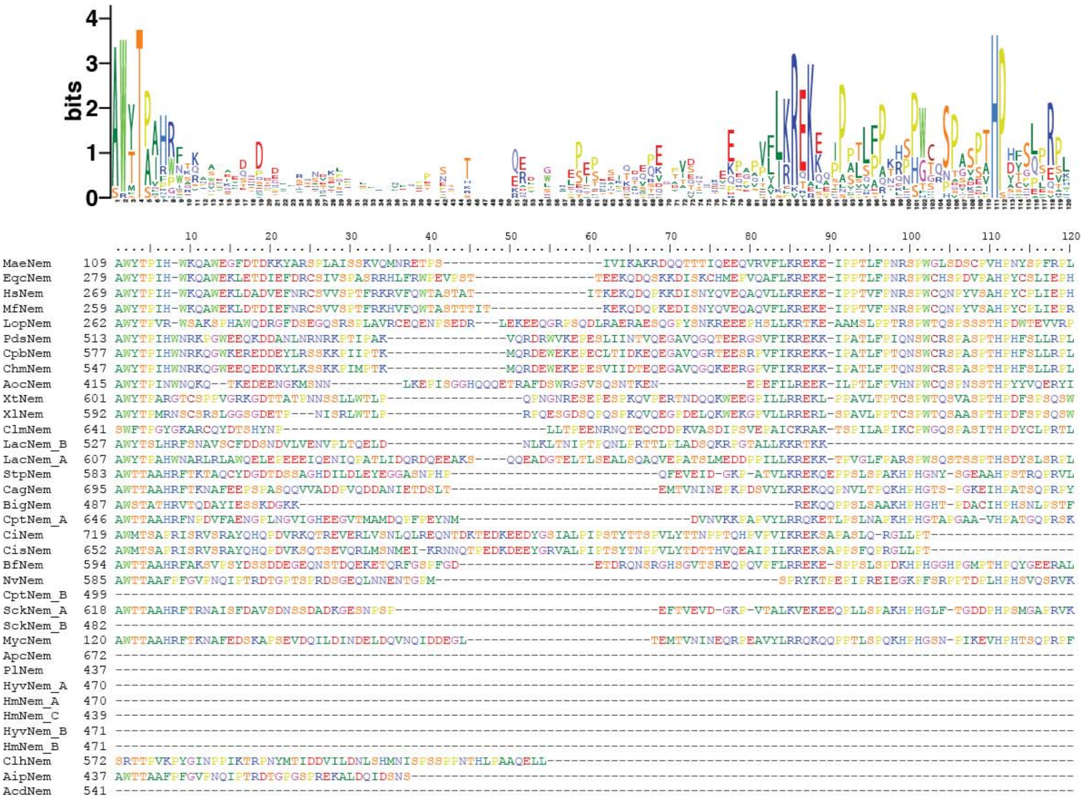

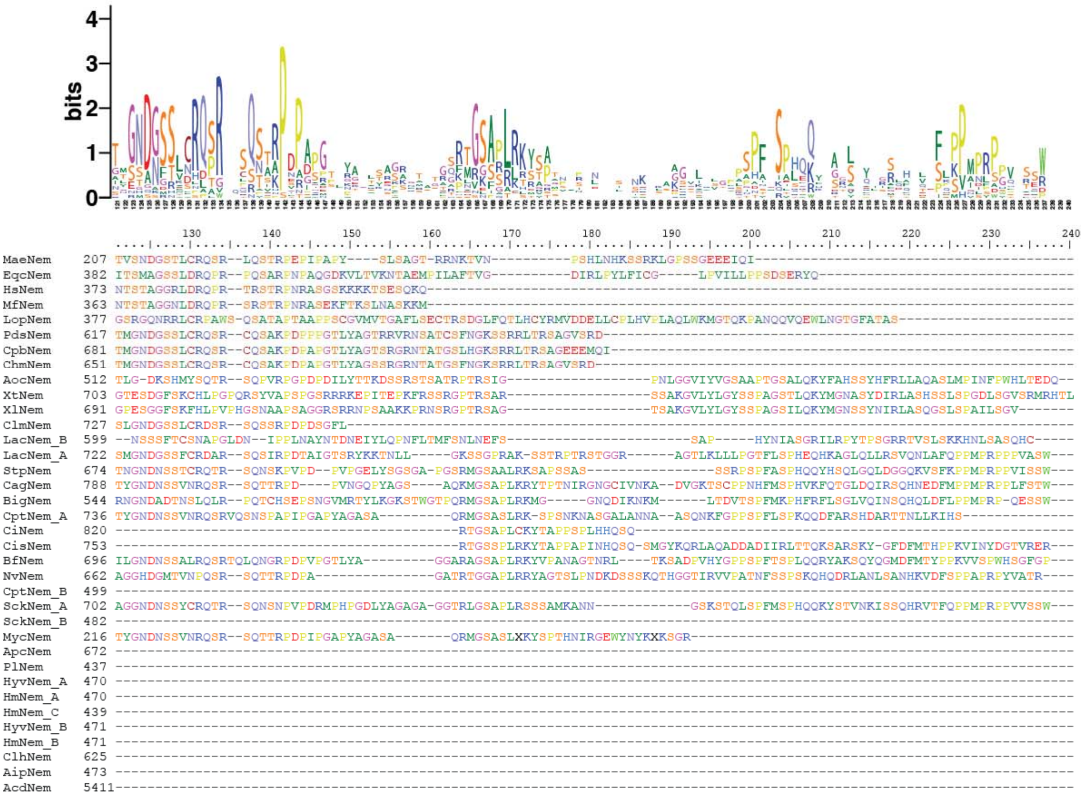

Supplementary Figure S6: The nematocilin tail domain is shown as WebLogo together with the alignment of nematocilin proteins. The numbers given in front of each sequence indicate the amino acid positions in the respective sequences. The CiNem, ApcNem, PlNem, ClhNem, AipNem and AcdNem sequences are incomplete, the CptNem_B, SckNem_B, HyvNem and HmNem nematocilins miss the IG and nematocilin-tail domains. The C-termini of the mammalian nematocilins are highly alternatively spliced.
